# Visual Rehabilitation in Chronic Cerebral Blindness: A Randomized Controlled Crossover Study

**DOI:** 10.3389/fneur.2016.00092

**Published:** 2016-06-17

**Authors:** Joris A. Elshout, Freekje van Asten, Carel B. Hoyng, Douwe P. Bergsma, Albert V. van den Berg

**Affiliations:** ^1^Section of Biophysics, Department of Cognitive Neuroscience, Donders Centre for Neuroscience, Donders Institute for Brain, Cognition, and Behaviour, Radboud University Medical Centre, Nijmegen, Netherlands; ^2^Department of Ophthalmology, Radboud University Medical Center, Nijmegen, Netherlands

**Keywords:** stroke, vision, rehabilitation, training, perimetry

## Abstract

The treatment of patients suffering from cerebral blindness following stroke is a topic of much recent interest. Several types of treatment are under investigation, such as substitution with prisms and compensation training of saccades. A third approach, aimed at vision restitution is controversial, as a proper controlled study design is missing. In the current study, 27 chronic stroke patients with homonymous visual field defects were trained at home with a visual training device. We used a discrimination task for two types of stimuli: a static point stimulus and a new optic flow-discontinuity stimulus. Using a randomized controlled crossover design, each patient received two successive training rounds, one with high contrast stimuli in their affected hemifield (test) and one round with low-contrast stimuli in their intact hemifield (control). Goldmann and Humphrey perimetry were performed at the start of the study and following each training round. In addition, reading performance was measured. Goldmann perimetry revealed a statistically significant reduction of the visual field defect after the test training, but not after the control training or after no intervention. For both training rounds combined, Humphrey perimetry revealed that the effect of a directed training (sensitivity change in trained hemifield) exceeded that of an undirected training (sensitivity change in untrained hemifield). The interaction between trained and tested hemifield was just above the threshold of significance (*p* = 0.058). Interestingly, reduction of the field defect assessed by Goldmann perimetry increases with the difference between defect size as measured by Humphrey and Goldmann perimetry prior to training. Moreover, improvement of visual sensitivity measured by Humphrey perimetry increases with the fraction of non-responsive elements (i.e., more relative field loss) in Humphrey perimetry prior to training. Reading speed revealed a significant improvement after training. Our findings demonstrate that our training can result in reduction of the visual field. Improved reading performance after defect training further supports the significance of our training for improvement in daily life activities.

## Introduction

Post-chiasmatic stroke may result in cerebral blindness. Patients with cerebral blindness suffer from a reduction of vision in the same part of the visual field of both eyes: a homonymous visual field defect (HVFD). Visual field defects vary in extent and quality, ranging from an absolute loss of vision to a relative field loss, in which vision is impaired but not completely absent. In addition, one can find regions within the blind hemifield that process visual information without awareness of the patient, resulting in “blindsight” (1–4). Such patients perform far above chance in a forced-choice visual discrimination task, while reporting no visual percept.

Homonymous visual field defects can seriously interfere with daily life activities like reading, recognition (of familiar persons, locations, or objects), mobility (loss of driving license, disorientation), and job security. It has a relatively high incidence; stroke occurs in about 4% of the population above the age of 65 in European countries (5) and about 30% of these include damage in the occipital region, causing cerebral blindness.

In about 40% of the patients, spontaneous recovery from HVFD occurs to variable extent within the first 3 months after stroke (6–9). Further, spontaneous improvement of the visual field becomes increasingly rare, and after 6 months, the patient enters the chronic phase of the hemianopia.

The treatment of patients suffering from blindness (hemianopia) following stroke is a topic of much recent interest, since a standard rehabilitation protocol is missing. Three rehabilitative approaches are examined, substitution with prisms, visual exploration therapy, and vision restitution (e.g., vision restitution training “VRT”). Therapies with prisms and visual exploration aim at substitution and compensation. While both approaches can be beneficial (10–14), the underlying problem, namely, the visual field defect itself is not targeted. Vision restitution aims to recover part of the blind field by extensively stimulating the border of the field defect, while patients maintain fixation on a central fixation point and make covert attention shifts toward the stimuli. The vision literature and perceptual learning literature strongly suggest that visual training may be beneficial for normal and impaired visual performers alike (15, 16).

Indeed, a number of studies have claimed that even in the chronic phase of stroke, part of the visual field can be improved through extensive visual training (17–24). Early observations of this kind (17, 18) have been criticized for technical reasons, i.e., inadequate control of eye movements (25, 26). Also, the lack of a proper control group (27, 28) to exclude both placebo effects and the possibility of spontaneous recovery has been noted. Previous studies applying different methods of vision training (20, 24, 29, 30) have collected a body of evidence that counters the first complaint. In the present study, we focus on the second objection that a large-scale study with a proper controlled study design is lacking.

Most previous studies selected chronic patients. Yet, some level of spontaneous recovery may occur, or factors independent of the *visual* training affect the extent of the visual field. To identify the contribution of non-intervention-related changes in effect parameters, one normally relies on a randomly attributed sham intervention. However, it is impossible to prevent the subjects from finding out by an eye movement that they receive sham training. Instead of a sham training of the defect, we offered a training of the intact side of the visual field as a control. Patients were selected for cortical damage in that part of the cortex, where the visual processing is limited to one hemisphere (i.e., the early visual areas up to V3). Therefore, we expected that training of the intact visual field would have no effect on the status of the damaged visual field and *vice versa*. This holds because early processing of the defect and the intact field occurs in opposite occipital hemispheres. Any improvement of the field defect by the control training we interpret as an a-specific effect of the training, raising the baseline against which the effect of the defect training is judged. We used Goldmann and Humphrey perimetry to assess training effects.

Finally, we observed that some particularly successful training studies in Huxlin’s lab (22–24) used visual motion for training rather than the appearance of static stimuli. Following up on this approach, we compared the effectiveness of training with visual point stimuli to optic flow stimuli that effectively stimulate the visual motion sensitive regions (31), which have been shown to reveal plastic changes following trauma early in life (32).

## Materials and Methods

### Study Design

This study was part of a larger project approved by the ethical committee CMO Arnhem–Nijmegen in conjunction with the 1964 Declaration of Helsinki. Forty stroke patients with visual field defects due to post-chiasmatic stroke were included following written informed consent. Patients throughout the Netherlands could sign up for our study voluntarily by filling in a form on our website. Patients in the chronic phase of stroke (>10 months post CVA) were included if they showed no signs of visual neglect (line bisection test). Patient age was between 18 and 75 years, and they were able to undergo (f)MRI scanning. The intake procedure further included a Goldmann perimetry measurement. All subjects had macular sparing of at least 2°.

To prevent selection bias, prior to the inclusion of the first patient, a training scheme for all patients was created using Matlab (Mathworks Inc.). For each cohort of 10 patients, numbers (J01–J40), the training stimuli (Flow or Point), and the order of training rounds [Test (defect) round first or Control (intact) round first] were randomly assigned to a patient number (Table S1 in Supplementary Material). The patients were included in order of registration on our website. Thus, the assignment of a patient to a patient number (with corresponding training scheme: stimulus type and the training order) was determined prior to the first inclusion, completely random and not based on selection.

We offered training in two parts (1) a test (defect training) and (2) a control (low-contrast training of the intact visual field). Following three cohorts of 10 patients, we modified the training procedure, because in some patients the control training reversed the increase of the visual field of the preceding defect training. Therefore, we adapted the design after the first three cohorts by targeting the affected hemifield exclusively and offered training per visual quadrant, using the untrained quadrant as control. Here, we report the training results of the first three cohorts, since the last cohort was not trained in their intact hemifield. The last cohort will be reported on a separate paper.

In all patients, training started 2–6 months after intake with baseline perimetry of the visual field defect. We applied both Humphrey (blinded) and Goldmann perimetry (not blinded, because of insufficient staffing).

Relative field defects may improve by training (detection thresholds decrease toward normal values assessed by Humphrey) but do not necessarily imply in a shift of the absolute border of the field defect, assessed by Goldmann. Thus, both perimetry methods describe the visual field (defect) in equally relevant but complementary ways.

**Figure 1:**
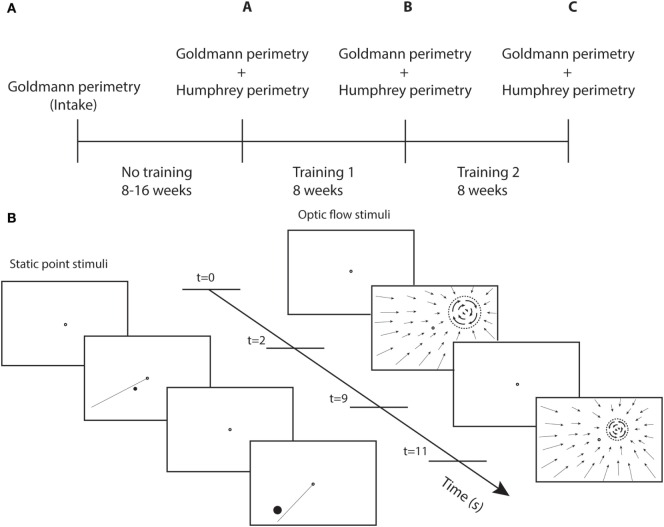
**Study design and training stimuli**. **(A)** Perimetry was performed at different time points. A-intake = effect no training; B-A = effect training 1; and C-B = effect training 2. **(B)** Sequence of screenshots for static point and optic flow discrimination task during two trials. Each trial starts with a single fixation point (2 s), followed by the stimulus (7 s). The dashed circle represent the black disk (itself invisible against the black background) on which a white optic flow pattern rotated clockwise or counterclockwise.

Perimetry was repeated following the first round of visual training (at least 40 h) and again following the second round of training (at least 40 h) (Figure 1A). The test and control training were evaluated for effectiveness by the difference between two succeeding perimetry assessments.

### Training

Each patient received a training unit at home to create a controlled training environment. This unit consists of a container, to be placed on a table, with a top cover and side covers to present a dark visual surround for the training stimuli with the exclusion of stray light. Mini Mac computer, keyboard, and mouse, a support with 24 ″ LED monitor, webcam, and chin/headrest were positioned inside the matte black container. Viewing distance was fixed at 40 cm. The subject’s face was indirectly lighted with a TL light for eye tracking with the web cam. The computer was prepared with eye tracking software, and training programs that were adjusted to the particular visual field defect of the patient.

Each patient served as its own control in a double (defect and intact) training paradigm. During defect training, high contrast stimuli (*C* > 0.9) were offered within the field defect along its border. During intact training, stimuli were presented within the intact field at about the same eccentricities as for the patient’s defect training (Table S1 in Supplementary Material). To offer a challenging training during the intact training, the stimulus contrast was reduced (*C* < 0.15).

We used two different types of training stimuli (Figure 1B). For both stimuli, the patient maintained fixation binocularly on a ring (diameter = 0.5°) at the center of the screen. During stimulus presentation (7 s), patients shifted attention covertly (i.e., without shifting eye fixation) toward the stimulus and responded using the keyboard. Only the fixation point was shown during the intertrial interval of 2 s.

Fifteen patients were trained with a static white point stimulus on a black screen. Point size was at least 0.2° in diameter (at 1° eccentricity) and was scaled with eccentricity:

(1) $$\text{scale(E)}=\left(0.0006{\text{E}}^{\wedge }2+0.0448\text{ E}+0.092\right)∕0.1374.$$

To cue the stimulated target location and to perform a discrimination task, a line was presented simultaneously with the point extending from the fixation target into the trained hemifield. The meridional angle of the line differed by 10° from the training point. The patient made a forced-choice response whether the point stimulus was presented clockwise or counterclockwise relative to the presented line.

The other group of 15 patients received an optic flow-discontinuity stimulus. Within the entire visual field, a pattern of flow was shown that contracted onto a training location within the visual field (white points on a dark screen). The stimulus to be discriminated was placed on a black disk (dashed circle Figure 1B) that covered the center of the contraction pattern, the diameter of which was eccentricity scaled with the same factor as for the point target. We used a minimal disk size of 1.7° at 1° eccentricity. The origin of the contraction pattern was the location cue for the flow discontinuity that had to be detected. The discontinuity stimulus (on the disk) was a flow pattern rotating clockwise or counterclockwise about the training location. The patient had to indicate the direction of rotation.

Throughout the training, fixation was monitored *via* a low-cost commercial webcam and eye tracking software available in the public domain (http://www.inference.phy.cam.ac.uk/opengazer/) that was adapted to supply eye position data to the training program (detailed description in Supplemental Material). Fixation position of both eyes during 0.5 s prior to stimulus onset was monitored (pretrial fixation). The fixation during the trial was compared to the pretrial mean (under assumption of proper fixation during pretrial). Our setup provided a horizontal accuracy of 0.2° and a vertical accuracy of 0.3°.

The length of one training session was on average 12 min (depending on the number of trials set per session and amount of fixation errors). The number of trials in a session ranged between 60 and 100, depending on the shape and quality of the visual field defect. The stimuli were randomly presented for each session.

The patients trained 1 h a day, 5 days a week during 8 weeks to complete at least 40 h of training per hemifield.

### Perimetry

Both types of perimetry were performed monocularly to the eye opposite to the affected hemifield. We applied the sita-fast 30-2 program of the Humphrey Field Analyzer II in a standardized way. Goldmann perimetry was performed using a white IV size stimulus with maximum luminance [4e = 1000 apostilb (asb) (≈318 cd/m^2^)] against a white background with a luminance of 31.5 asb. Fixation was continuously monitored *via* the spyglass by the experimenter and checked at random occasions using the Heijl–Krakau method (33) (blind spot localization) that is also used during Humphrey perimetry.

Mean sensitivity of Humphrey perimetry was calculated for each hemifield separately. Thus, we were able to investigate the change in sensitivity through directed training (i.e., change in sensitivity of the trained hemifield) and undirected training (i.e., change in sensitivity of the untrained hemifield) for both defect and intact training. Furthermore, we created isopters based on the Humphrey data and compared them with the Goldmann isopters. We expressed the visual field border change in Equivalent Cortical Surface Gain (ECSG) (21).

### Equivalent Cortical Surface Gain

The central 30° of the Goldmann perimetry was compared with the 30-2 program of the Humphrey perimetry session. To describe changes in Goldmann visual field isopters (i.e., border shifts), we calculated the ECSG from the bordershift. ECSG transforms the increase of the eccentricity of the border into an equivalent measure of millimeter cortex shift. This is done using recently published fMRI data of cortical scaling in area V1 (34). We converted each perimetric map into a pixelmap, with weights for each pixel according to its eccentricity (E) from the foveal projection in the map:

(2) W(E)=21∕E.

**Figure 2:**
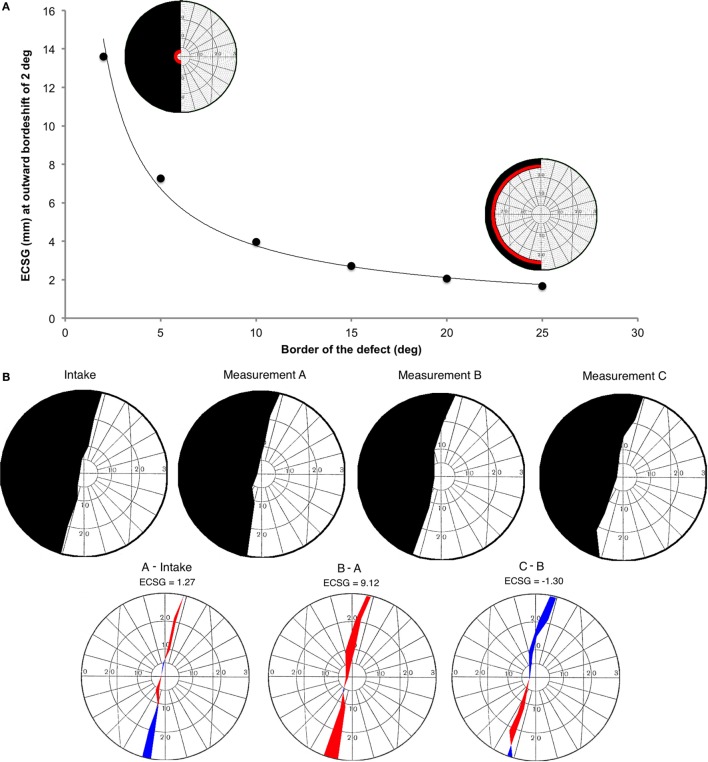
**Introduction of a new method to describe visual field changes in terms of equivalent cortical surface gain (ECSG) based on the cortical magnification factor**. **(A)** mm ECSG for 2° of field increase across the entire hemifield, starting at a pre-training defect border of 2°, 5°, 10°, 15°, 20°, and 25° eccentricity. The corresponding area of a 2° field increase (red) is shown for a defect border starting at 2° and 25°, respectively. **(B)** Goldmann perimetry during intake, prior to training (measurement A), after 40 h of training the affected hemifield (measurement B) and after 40 h training the intact hemifield (measurement C) in one subject (J22). The graphs below show the visual field change, when no intervention was done (A-Intake), for defect training (B-A) and for intact training (C-B). Red represent field increase and blue represent field decrease. The corresponding ECSG values are 1.27, 9.12, and −1.30, respectively. Note that, this patient with right occipital lesion shows a visual field defect crossing the vertical midline. This is reproduced in all four Goldmann measurements and also visible in all three Humphrey measurements.

This function is derived from the eccentricity of the voxel’s receptive field as a function of the distance within area V1, as reported by Wu et al. (34) (their Figure 4). W(E) describes the derivative to retinal eccentricity of the inverse of their function. We then summed across the hemifield, the weighted pixel values that were located in between the defect borders of the pre- and post-training perimetric maps. We assigned a positive weight when the pixel was located in a sector where the field defect was reduced and a negative weight when the defect was increased (Figure 2B). Dividing the integral by π, one obtains the average (across meridional angle) cortical shift in the eccentricity direction (ECSG, units: millimeter) at the human primary visual cortex.

We calculated the millimeter-shift on the cortex for a simulated reduction of the eccentricity of the field defect by 2° in the entire hemifield, to check validity of the ECSG. We did so for defect borders at 2°, 5°, 10°, 15°, 20°, and 25° eccentricity (Figure 2A). These cortical shifts corresponded to the fitted function of cortical location vs. eccentricity, as reported by Wu et al. ECSG, in essence, applies the cortical magnification factor (CMF) to the border shift in the visual field. Performance on many visual tasks follows the CMF, meaning that 1° of recovered vision at the center of the visual field implies a larger performance gain than 1° border shift in the periphery. Thus, we quantified the notion that more functional recovery occurs when 1° of central vision is recovered than 1° of peripheral vision.

### Comparing Isopters of Goldmann and Humphrey Perimeters Prior to Training

Prior to the first training (Figure 5A), the 0 dB Humphrey threshold contour (H_0_: connecting the border locations where brightest flashes were not detected) clearly defined a more extensive defect than was obtained from the Goldmann perimetry in most patients. In contrast, the contour with just responsive locations [i.e., non-zero dB threshold (H_1_)] showed a smaller defect than the Goldmann in most patients. Taken together, this suggests that Goldmann perimetry (ECSG) and the average of the two Humphrey perimetry contours (H_mean_) established roughly the same defect border at the outset. If so, the border shift (ECSG_H0−G_) between Goldmann contour (G) and the H_0_ contour should be about the same and opposite as the border shift (ECSG_H1−G_) between the G contour and the H_1_ contour. Because the ECSG measure rests on summation of the same non-linear transformation to eccentricity for all three maps (G, H_0_, and H_1_), the following holds:

(3) $$0.5\times \text{(ECS}{\text{G}}_{\text{H0-G}}+\text{ ECS}{\text{G}}_{\text{H1-G}}\text{)}=\text{ECS}{\text{G}}_{\text{Hmean}}-\text{ECS}{\text{G}}_{\text{G}}.$$

We calculated the difference between the defect border estimated from the “average” of the Humphrey contours and the Goldmann defect border (H_mean_: dashed line in Figure 5A) from the left hand side of above equation.

### Reading Test

Two different texts (15-point Arial font; between 88 and 165 words) were used for each patient to assess reading speed prior to training and, following defect and intact training. A chin rest stabilized the patients’ head at a distance of 50 cm. Reading performance was assessed using a head mounted eye tracker (Eyelink II, SR Research, Ontario, Canada), while patients read the texts for themselves. Reading speed [words per minute (WPM)] was calculated from the time between first and last saccade. Effect of training on reading performance was calculated as percentage increase in WPM:

(4) $$\text{100 }\times \left(\text{WP}{\text{M}}_{\text{post}}\text{/WP}{\text{M}}_{\text{pre}}-\text{1}\right).$$

### Statistical Analysis

Repeated measures ANOVAs were used to test for any interaction effects (flow vs. point, defect training first vs. intact training first). For Goldmann perimetry, we used *post hoc* Wilcoxon paired samples tests to compare the effect of the different training rounds with no intervention. For Humphrey perimetry, we used *post hoc* Wilcoxon paired samples tests to compare the effect of the directed training with undirected training. For Humphrey perimetry and reading speed, we used a Wilcoxon test with a hypothesized median of 0, as Humphrey perimetry and the reading task were not done during intake. We used regression analyses to study linear relationships between effect measures. We considered a *p* < 0.05 as statistically significant. SPSS V.20.0 (SPSS Inc., Chicago, IL, USA) was used for analyses.

## Results

Three patients dropped out during the first training round for personal reasons and were excluded from analyses. Remaining 27 patients were used for analyses [5 women (18.5%), 22 men (81.5%); mean age 51.2 years, range 29–74]. Fourteen patients suffered from a left-sided visual field defect, and thirteen had a right-sided visual field defect (detailed demographics in Table S1 in Supplementary Material). Fixation during training at home was variable among patients. On average, patients maintained stable fixation within 2° from the fixation point in about 88% of the trials (Figure S1 in Supplementary Material).

Of these 27 patients, we could not perform the ECSG analysis in two patients, for whom a complex relative visual field defect precluded definition of a Goldmann isopter. We did analyze their Humphrey perimetry and their fixation data from the training at home. All Goldmann perimetry data of the other patients were considered reliable following standard procedure (detailed description in Supplementary Material). Five of the 27 patients were excluded from the analysis per hemifield, since at least one of the three Humphrey measurements they performed was unreliable (>20% false detections during blind spot probing). Two of them did perform reliable Humphrey measurements during the defect training round.

### Goldmann Perimetry

Goldmann perimetry was used to detect a possible shift of the absolute field *border*.

We measured border shift following no intervention (the difference between the A measurement and intake) and following intact training as controls.

**Figure 3:**
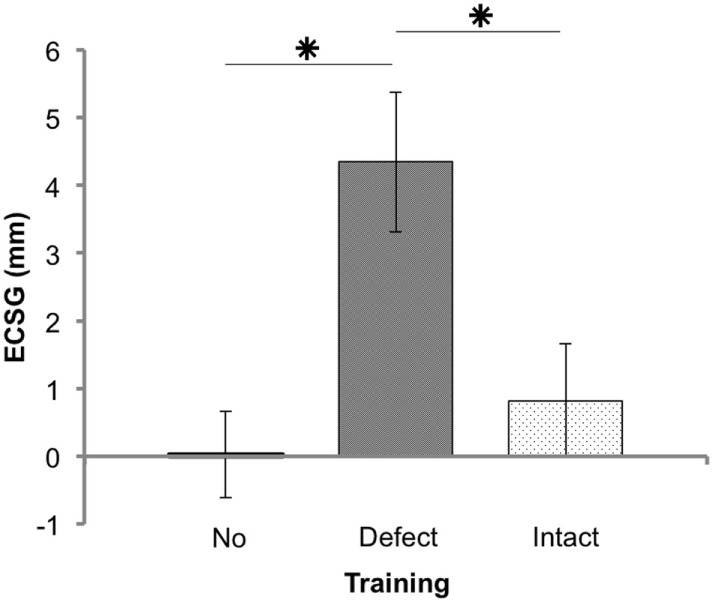
**Results of Goldmann perimetry following each training round**. Defect reduction measured with Goldmann perimetry (ECSG: mean ± SEM for 25 patients) following no intervention, defect training, and intact training. The effects of the training rounds were assessed with respect to the preceding perimetry measurement. Note that, patient J18 and J23 were excluded for this analysis.

There was no significant border shift without an intervention (*p* = 0.600, Figure 3). We found a significant interaction between the factors, trained hemifield and training order (F(1, 21) = 6.13, *p* = 0.020, see Figure S2A in Supplementary Material). Also, there was a main effect of trained hemifield (F(1, 21) = 5.93, *p* = 0.024). There was no interaction with stimulus type (F(1, 21) = 0.155, *p* = 0.698). When patients start training their intact visual field, both training rounds achieved about a similar reduction of the field defect (ECSG ≈2 mm). In contrast, training the visual field defect first yielded an increase of ECSG that was partially reversed by the ensuing intact training (ECSG < 0, Figure S2A in Supplementary Material).

Training the defect side resulted in a larger Goldmann ECSG (M 4.34, SEM 1.03) than no intervention ECSG (M 0.03, SEM 0.67) and intact training ECSG (M 0.82, SEM 0.84). Both differences were significant (*Z* = −3.081, *p* = 0.002 and *Z* = −2.005, *p* = 0.045, respectively). Training the intact side elicited no significant effect compared to no intervention (*Z* = −0.834, *p* = 0.404). Together, these data show a larger shift of the border by defect training than following no intervention or by intact training.

### Humphrey Perimetry

Humphrey perimetry was used to study how the defect and intact training affect the visual *sensitivity* in both hemifields.

We assessed the effect of each training on the trained and untrained hemifield separately, from the mean visual sensitivity per hemifield. Thus, we have two control measures, the undirected training during defect training (i.e., sensitivity change in intact hemifield) and the undirected training during intact training (i.e., sensitivity change in defect hemifield).

**Figure 4:**
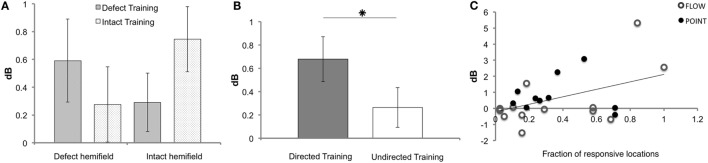
**Results of Humphrey perimetry following each training round**. **(A)** Mean sensitivity change in dB (± SEM) per hemifield. **(B)** Mean sensitivity change in dB (± SEM) for directed and undirected training combined for both hemifields. **(C)** Sensitivity increase measured with Humphrey perimetry (dB) after directed defect training as a function of number of non-responsive elements prior to training (A measurement). Note that patients J09, J10, J13, J14, and J22 were excluded for the Humphrey hemifield analysis **(A)**, because this analysis requires three measurements with adequate fixation. For J14 and J22, data were included for the defect training round analyses **(B,C)**, because their pre- and post-defect training perimetry was reliable.

Remarkably, we observed in a subset of the patients that not only the defect training improved the sensitivity of the defect hemifield but also that the intact training improved the sensitivity in the “intact” hemifield. Correspondingly, we found a near significant interaction between measured hemifield (defect vs. intact) and trained hemifield (defect training vs. intact training) (F(1, 18) = 4.104, *p* = 0.058). There was no main effect of trained hemifield (F(1, 18) = 0.635, *p* = 0.436) (Figure 4A).

As shown in Figure 4B, the overall effect of the directed training exceeded that of undirected training (*Z* = −2.534, *p* = 0.011). Both training rounds combined showed a significant increase of sensitivity for directed training (M 0.68, SEM 0.19), but not following undirected training (M 0.26, SEM 0.17) (*t* = 3.505, *p* = 0.001 and *t* = 1.546, *p* = 0.129, respectively). Note that, our study lacked the power to conclude that the defect training or intact training by themselves are more effective for directed than undirected training.

### Predicting Training Effects from Pre-Training Field Status

Our patients varied considerably in extent of the field defect and the depth of their sensitivity reduction. Also, the effect of the training varied considerably among patients. We wondered whether pre-training field status might be predictive of the effectiveness of the training.

First, we assessed the fraction of responsive locations in the affected hemifield prior to the first training round (locations with >0 dB threshold), as we reasoned that flash sensitivity might increase more if the absolute blind field is smaller (i.e., larger relative field defect). Indeed, a significant linear relation was found between this measure and Humphrey sensitivity increase in the affected field for *defect* training [beta = 0.457, *t*(22) = 2.41, *p* = 0.025] (Figure 4C). This linear relationship was not found for the affected hemifield after intact training [beta = 0.341, *t*(22) = −1.703, *p* = 0.103] and the number of responsive locations.

**Figure 5:**
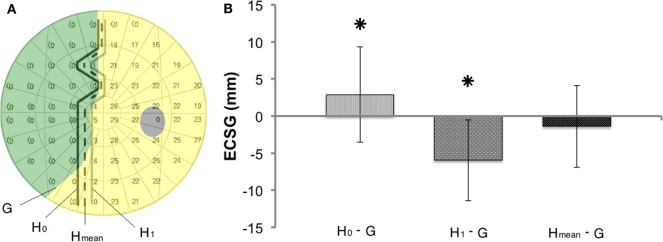
**Method to compare shifts in the visual field border based on Goldmann and Humphrey perimetry (A) Definitions of the border of the defect (G, H_0_, H_1_, and H_mean_), using the data of one subject (J13)**. **(B)** Border difference between Goldmann (G) and Humphrey perimetry prior to training (H_0_ or H_1_ or H_mean_). Mean ± SD for all patients. Prior to training H_mean_ defect border corresponds best to the Goldmann border, as it does not deviate from 0 in contrast to H_0−G_ (*p* = 0.012) and H_1−G_ (*p* = 0.000).

In our search for a similarly predictive measure for Goldmann perimetry, we established visual field isopters based on the Humphrey data. As explained before, we took for each patient, the isopter of the 0 dB contour line and the >0 dB contour line (Figure 5A) to establish its mean. We compared this averaged Humphrey isopter to the Goldmann isopter for the data collected at start of the study (measurement A). Averaged across the group of 22 patients with complete data, the ECSG difference was small (H_mean–G_ = −1.39, SD = 5.5) and not significant (*p* = 0.426). Thus, at the outset of the training, the absolute field border established by Humphrey and Goldmann perimetry was about equal on average. However, there was a considerable variation between patients ranging from Humphrey field extending beyond the borders of the Goldmann perimetry to the other way around. We wondered whether this border difference between the two perimetry measures in a single patient at the outset could predict its training success with regard to Goldmann perimetry?

**Figure 6:**
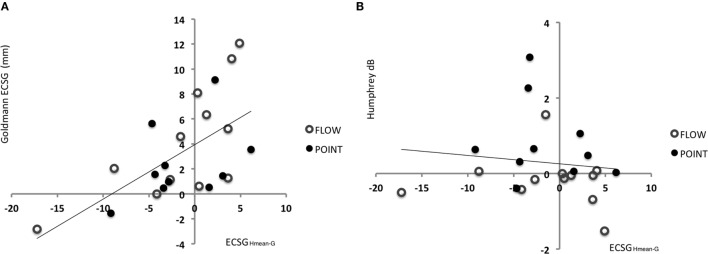
**Indication for recovery potential based on the difference between Goldmann and Humphrey perimetry *prior* to training**. **(A)** Regression between defect reduction measured with Goldmann perimetry (ECSG) after defect training and the difference between the Humphrey and Goldmann perimetry prior to the training (A measurement: border of G – border of H_mean_ expressed in ECSG). Clearly, the defect reduction was linearly related to the border difference between Humphrey and Goldmann perimetry prior to training. Patients’ border shift by Goldmann perimetry equals 3.9405 + 0.436 ECSG_Hmean−G_. **(B)** The same analysis for the sensitivity increase (dB) in the affected hemifield by defect training.

Accordingly, we used the difference between Goldmann and Humphrey borders prior to training (ECSG_Hmean–G_) as a measure of pre-training field status. We regressed this measure with the ECSG established from the Goldmann perimetry after *defect training* (Figure 6A). Across the patient group, a significant regression was found between these measures of pre-training field status and defect training result: beta = 0.607, *t*(20) = 3.41, *p* = 0.003. In contrast, the pre-training field status did not predict the effect of *defect training* on Humphrey sensitivity [Figure 6B: beta = −0.122, *t*(20) = −0.551, *p* = 0.587] and the effect of *intact training* on Goldmann perimetry of the damaged field.

### Reading

Reading performance was collected for all 27 patients. Due to technical failure, data of one patient was lost. There were significant interactions between the factors, trained hemifield and training order (F(1, 22) = 6.306, *p* = 0.020) and between the factors, trained hemifield and stimulus type (F(1, 22) = 4.901, *p* = 0.038) (Figures S2B,C in Supplementary Material).

**Figure 7:**
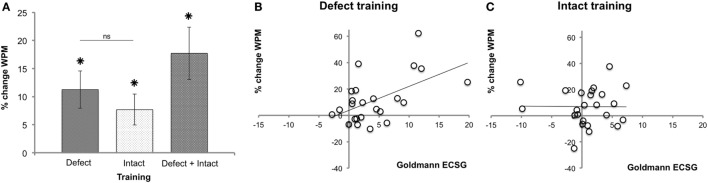
**Reading performance following each training round**. **(A)** Reading speed improvement (mean ± SEM, *n* = 26). **(B)** Regression between reading performance and defect reduction measured with Goldmann perimetry (ECSG) after defect training (*n* = 24). Clearly, the defect reduction was linearly related to the reading improvement after defect training (% change WPM = 3.78 + 1.82 Goldmann ECSG). **(C)** The same analysis for the intact training revealed no such relation.

Figure 7A shows the main effects on reading speed. After both defect (M 11.26%, SEM 3.34) and intact training (M 7.70%, SEM 2.75), reading speed was significantly (*p* = 0.002 and *p* = 0.011, respectively) improved. We found no effect difference between defect and intact training rounds (*p* = 0.551).

The Goldmann ECSG and the increase in reading speed after defect training were linearly related (Figure 7B): beta = 0.542, *t*(22) = 3.021, *p* = 0.006. Reading showed no such linear increase with the intact training [beta = −0.01, *t*(23) = −0.046, *p* = 0.963] (Figure 7C). Thus, although both defect and intact training increased reading speed, only after defect training the improvement in reading speed was positively related to the increase in Goldmann ECSG. Reading speed showed no such linear relation with Humphrey perimetry (*p* > 0.518).

## Discussion

Our randomized controlled crossover study demonstrates significant improvements of the visual field of patients with cerebral blindness by our visual discrimination training. Our works add to previous non-RCT studies (20, 22, 24, 29, 30) that visual training effects are specific for a *directed* visual training and not visual training *per se*.

Goldmann (defect border) perimetry showed a significant reduction of the field defect by directed training. In contrast, training of the opposite hemifield did not reduce the field defect. In addition, when no intervention was done in a 2-month period between intake and the first training round, no change of the defect border was observed through Goldmann perimetry. Humphrey perimetry (dB sensitivity) partially supported the Goldmann results; the effect of a directed training exceeded that of an undirected training for both training rounds combined but lacked the power to verify this for each hemifield separately. Note that, the statistical power of the Humphrey results is relatively low given the moderate effect size [(1−β) = 0.50] in this relatively small group, in contrast to the Goldmann results [effect size = 0.68; power (1−β) = 0.96]. Nevertheless, these outcomes indicate that even a relatively brief, intense visual training period of the defect side (8 weeks) results in a small but significant improvement of visual performance in this group of patients.

One could object that the Goldmann results could be influenced by unintentional experimenter biases. However, both after defect and intact training positive and negative results were found. Moreover, the effects were not (always) along the entire border of the defect but could be present for only a specific region. These irregular and unpredictable effect outcomes appear inconsistent with systematic experimenter bias. Finally, the Humphrey data showed a similar pattern as the Goldmann and confirmed that only a directed training could potentially improve the visual field.

Could the effects of our training just be a modulation of an ongoing spontaneous recovery? According to Zhang et al., spontaneous recovery can occur up to about 6 months after stroke (8). In all our patients, the training started at least 4 months later (mean = 23 months poststroke). Indeed, we found no change in Goldmann ECSG between intake and the start of the training, indicating that without intervention the state of the visual field was stable during at least 2 months prior to training. Thus, we feel justified to conclude that the small but significant improvements we found are not induced by spontaneous recovery.

To our knowledge, we investigated for the first time, fixation data, during visual training at home. Calibration procedure turned out to be difficult for some patients causing a loss of fixation data for about 40% of the trials. Nevertheless, fixation turned out to be surprisingly accurate. About ~90% of the time, patients maintained fixation within our criterion of 2° from the fixation point’s center (Figure S1 in Supplementary Material). This shows that fixation control during visual training at home is feasible, and that fixation registration *via* a low-cost webcam can help to guide the patient during rehabilitation by visual training.

Overall, in accordance with numerous older studies (17–21, 29, 30), our critical analysis, including a control training, supports previous work, including sophisticated laboratory studies (22, 24), that a visual training can reduce the visual field defect in the chronic phase of stroke, even for patients who train at home.

One study used a control training evoking eye movements to stimuli near the fovea (17). Patients with post-chiasmatic lesions only showed field improvement after test and not following that control training (17). This is in line with our findings; however, the effect of that study amounted to about 1 SEM of the performance prior to training. We report a much larger effect of >2 SEM for both types of perimetry. Our work extends their result further, because Kasten et al.’s control shows that merely presenting small visual stimuli near the fovea is not an effective training stimulus. In our study, patients that followed the optic flow training did receive visual stimulation in their entire visual field during both control and test training. We did not find an effect of training stimulus in the analysis of Humphrey perimetry (dB sensitivity measure) or the Goldmann perimetry (ECSG measure of defect border position), indicating that extensive visual stimulation of the defect is not an effective training by itself but only in combination with a task that directs visual attention to the defect. However, these results must be interpreted with caution, since analyzing these two factors in a full factorial design result in relatively small groups.

Previously, peripheral motion stimulation revealed larger reduction of patients’ field defect than the standard point stimuli in a detection task (35). We found no support that optic flow is more effective than static point stimulation in our discrimination task. Following training with motion stimuli Das et al. found marked improvements in patients suffering from cortical blindness (occasionally up to restoration to normal performance level) in motion discrimination (22–24). Comparing our work quantitatively to the motion-training studies of Huxlin’s lab (22–24) is difficult. We used different flow patterns, and we trained many more different locations (>60 per hemifield for each patient) without selection for high potential locations (e.g., locations with blind sight) prior to training, reducing the amount of training hours of such “optimal” locations. We reported averaged improvements per hemifield instead of selecting a region of interest with high potential. All our choices lead to less marked sensitivity increases, as we take the whole field defect in the central 30° into account for sensitivity decrease, including many locations that were marginally trained. Nevertheless, we find convincing evidence that training at home during 8 weeks is beneficial with an improvement of 0.5–1 dB, provided it is directed at the defect. By way of comparison: a decrease between 0.5 and 1.5 dB per year is considered a moderate progression in glaucoma (36).

Furthermore, the improvement in reading speed supports the significance of our visual training. Both types of training result in a general improvement of the reading speed. Importantly, only the defect training also results in a linear relationship between the magnitude of the field recovery and the improvement of reading speed. This suggests that increased reading speed results from the extent of field recovery or both grow in common from another effect of the defect training. Our observations are in contrast to the task specific results found by Schuett et al. They found significantly higher improvements in reading following a reading training compared to exploration training (13). In addition, another study did not find reading improvements following either exploration training or attention training (12). In line with our results, other studies using training aimed at vision restitution also report improved reading performance (20, 21, 37).

Gall and Sabel did not find a relation between reading performance and field change after restitution training based on high-resolution perimetry (37). Remarkably, we did find correlations between effect measures. First, the interaction between training and training order for reading was identical as that for Goldmann perimetry (Figure S2 in Supplementary Material). Second, we found a correlation between the reading improvement and Goldmann ECSG after defect but not after intact training. While we did not train a reading task, our training can contribute to an enlarged functional reading window.

In conclusion, in a population of 25 patients with HVFDs, our visual discrimination training reduced the field defect. Interestingly, the individual outcome of the training was not completely random but appeared related to the status of the visual field at the training’s outset, when both types of perimetry were compared. When the blind field was larger according to Goldmann perimetry than according to Humphrey perimetry, the gain of the visual field following defect training was larger in the Goldmann perimeter. Likewise, using Humphrey perimetry, sensitivity increase by defect training was larger when prior to the training more locations were affected but not completely blind (>0 dB thresholds). These correlations were only found after the defect and not intact training. Both pre-training measures could indicate which HVFD patients might benefit most from this rehabilitation method, potentially increasing the yield. To investigate the surplus value of training with motion stimuli compared to static point stimuli, additional studies with larger groups are required. Larger groups will also increase the statistical power, given the moderate effect size of the Humphrey results found in this study.

Training of residual functions for other disabilities following stroke, e.g., aphasia and motor-defects, are generally accepted practices. In contrast, claims to ameliorate cerebral blindness by a rehabilitation approach have been under debate. Inspired by the perceptual learning field, there is a growing interest to treat central visual disorders like amblyopia (38) by visual training. Similarly, visual training aimed at vision restitution may become a tool for the treatment of HVFDs, in addition to compensatory and substitution approaches (39).

## Author Contributions

JE, DB, and AB contributed to experimental design. JE and AB contributed to data analysis and data interpretation. JE, FA, CH, and DB contributed to data acquisition and data interpretation. All authors were involved in the writing of the manuscript.

## Conflict of Interest Statement

The authors declare that the research was conducted in the absence of any commercial or financial relationships that could be construed as a potential conflict of interest.
