# Supplementary material for: Visual Rehabilitation in Chronic Cerebral Blindness: A Randomized Controlled Crossover Study
Source: Front Neurol. 2016 Jun 17;7:92. doi: 10.3389/fneur.2016.00092 (PMC4911356; doi:10.3389/fneur.2016.00092)
Supplement: Supplementary file 1 [file datasheet_1.docx]

**Visual rehabilitation in chronic cerebral blindness: a randomized controlled crossover study**

J.A. Elshout^1^, F. van Asten^2^, C.B. Hoyng^2^, D.P. Bergsma^1^, A.V. van den Berg^1^

**^1^**Department of Cognitive Neuroscience, Section of Biophysics, Donders Centre for Neuroscience, Donders Institute for Brain, Cognition, and Behaviour, Radboud university medical centre, Nijmegen, the Netherlands

**^2^**Department of Ophthalmology, Radboud university medical centre, Nijmegen, the Netherlands

**Supplemental material**

**Fixation data during visual training at home**

Following the regular calibration procedure (see Fig. S1 A) as used for all patients, point stimuli were presented on the horizontal- and vertical-axis at 1, 2 or 3 degrees from the center in a sequence of trials to five healthy subjects. The subjects were asked to fixate the eccentric stimulus point on appearance until it disappeared (4 seconds) after which they refixated the central point. The last 25 data points of each trial (~ 2.5 seconds) were used to determine the SD of the tracker signal. We report tracker accuracy from SD in horizontal and vertical directions, averaged across subjects and fixation locations.

To investigate how well the patient could execute the training procedure at home we analyzed the eyetracking data of all trials collected via the webcam inside the training unit. Prior to each training, the patient performed a calibration procedure that allowed the software to establish from the camera image the fixated pixel on the screen, by taking into account small head movements that were tracked from characteristic points on the face. The training software calculated gaze direction errors (criterion: >2 deg deviation from the pretrial gaze direction in any epoch >100 ms during the trial). The trials during which fixation was lost were repeated once at the end of the session.

Across all trials, subjects and both training rounds, we collected on average 61% of usable webcam data for further analysis (Fig. S1 B). We calculated the percentage of trials with good fixation. The mean fixation loss was 12%, meaning that in 88% of the cases patients were able to keep fixation in a window of 4 by 4 degrees during stimulus presentation (Fig. S1 C).


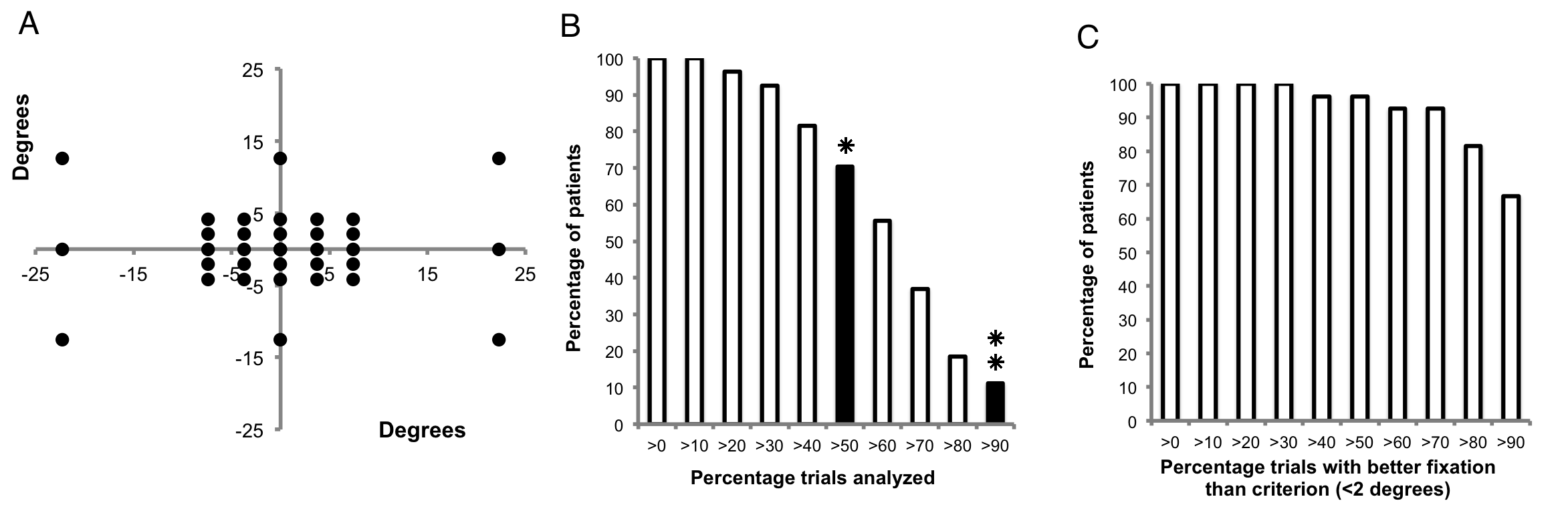


Figure S1. Webcam based control of eye fixation. Fixation data were collected during visual training at home using a webcam for a total of 27 patients. (**A**) Pattern of calibration points optimized for accuracy around the fixation point. (**B**) Gaze data-collection could fail due to inappropriate calibration by the patient resulting in loss of trial data. Cumulative number of patients that allowed eye fixation analysis in at least K percent of the trials. Patients with more than 50% of the trials analyzed are marked with one star in supplementary table S1. Patients with more than 90% of the trials analyzed are marked with two stars in supplementary table S1. (**C**) Number of patients (cumulative) with better eye fixation than the criterion in at least N percent of the trials that were available for analysis.

**Goldmann perimetry procedure**

Goldmann perimetry uses a continuous moving light stimulus along a trajectory approximately perpendicular to the border to characterize the visual field defect. It is a sensitive and efficient method to assess the location of the absolute defect. However, because it is not an automated procedure, the measured field is dependent on the examiners skills.

To optimize test reliability a trained examiner used the following procedure:

1. Carefully instruct the patient to fixate on the central fixation point
2. About twenty-five trajectories were probed in random order. This was repeated three times.
3. Fixation was *continuously* controlled during *each* trial via the spyglass and the trial was repeated when a saccade of the patient was observed.
4. The blind spot was measured and randomly probed every 5-10th trial. A positive response by the patient (which occurred rarely; in less than 20% of the cases) was followed by an instruction to fixate carefully.

Patients with serious fixation problems (assessed during intake) were not included in the study since both the training itself and the field assessments required adequate fixation.

**Interaction effects Goldmann and reading performance**


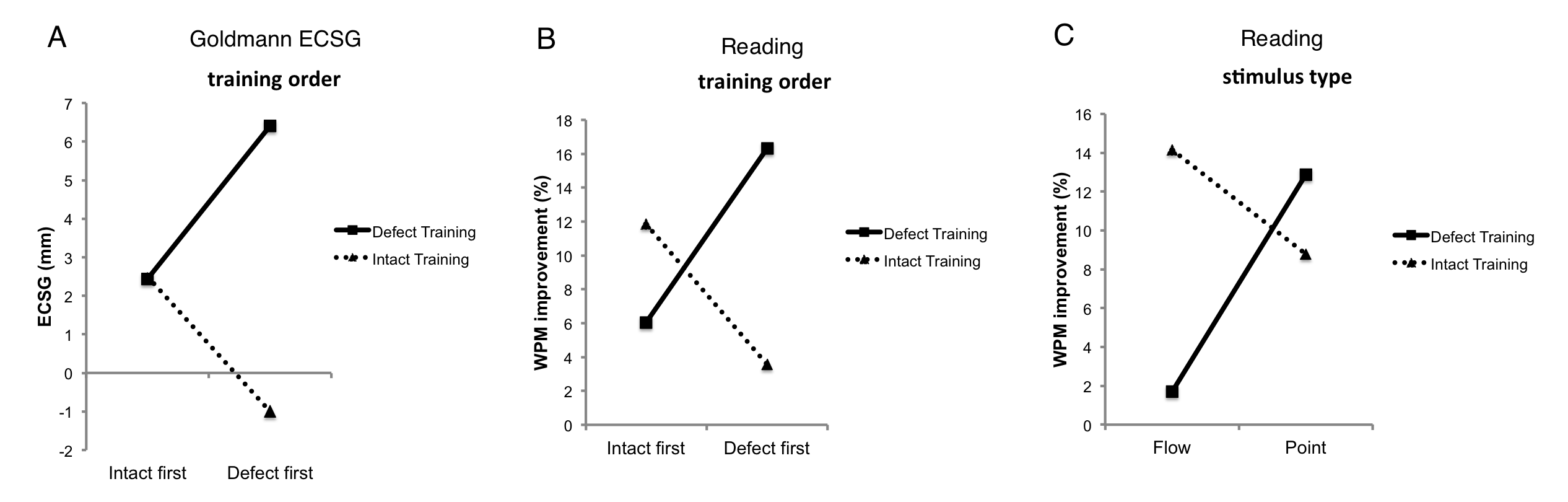


Figure S2. Interaction effects. (**A**) Interaction between trained hemifield and the order of the two training rounds in Goldmann perimetry. (**B**) Interaction between trained hemifield and the order of the two training rounds in reading performance. (**C**) Interaction between trained hemifield and stimulus type in reading performance.

**Table S1:** Patient demographics

| Subject | Sex | Age (years) | Time since lesion (months) | Field defect | Lesion | Cause | Training paradigm^a^ | Training locations |
| --- | --- | --- | --- | --- | --- | --- | --- | --- |
| J01* | M | 66 | 27 | Hemi-L | right occipital/parietal cortex | Ischemic stroke | FID | 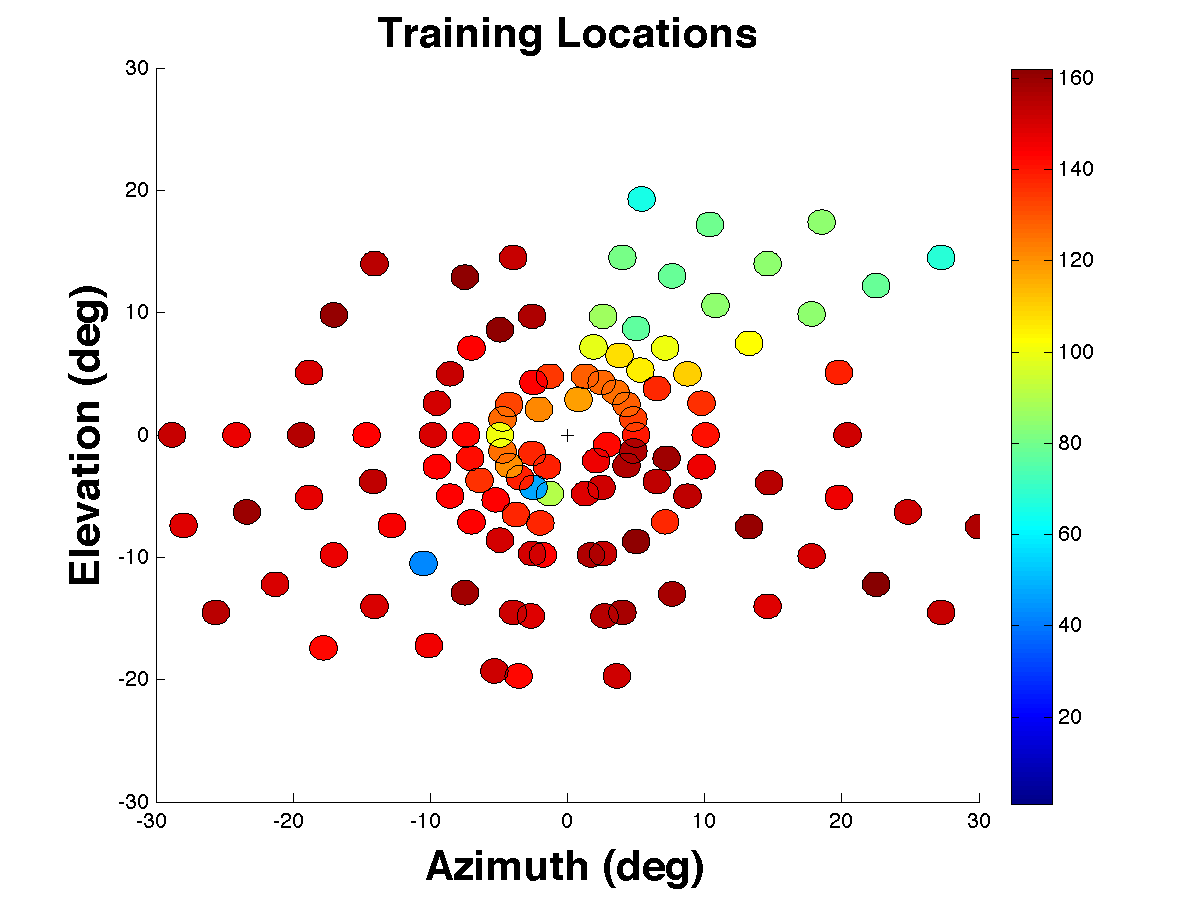 |
| J02* | M | 61 | 12 | Scot-L | right occipital cortex | Ischemic stroke | PDI | 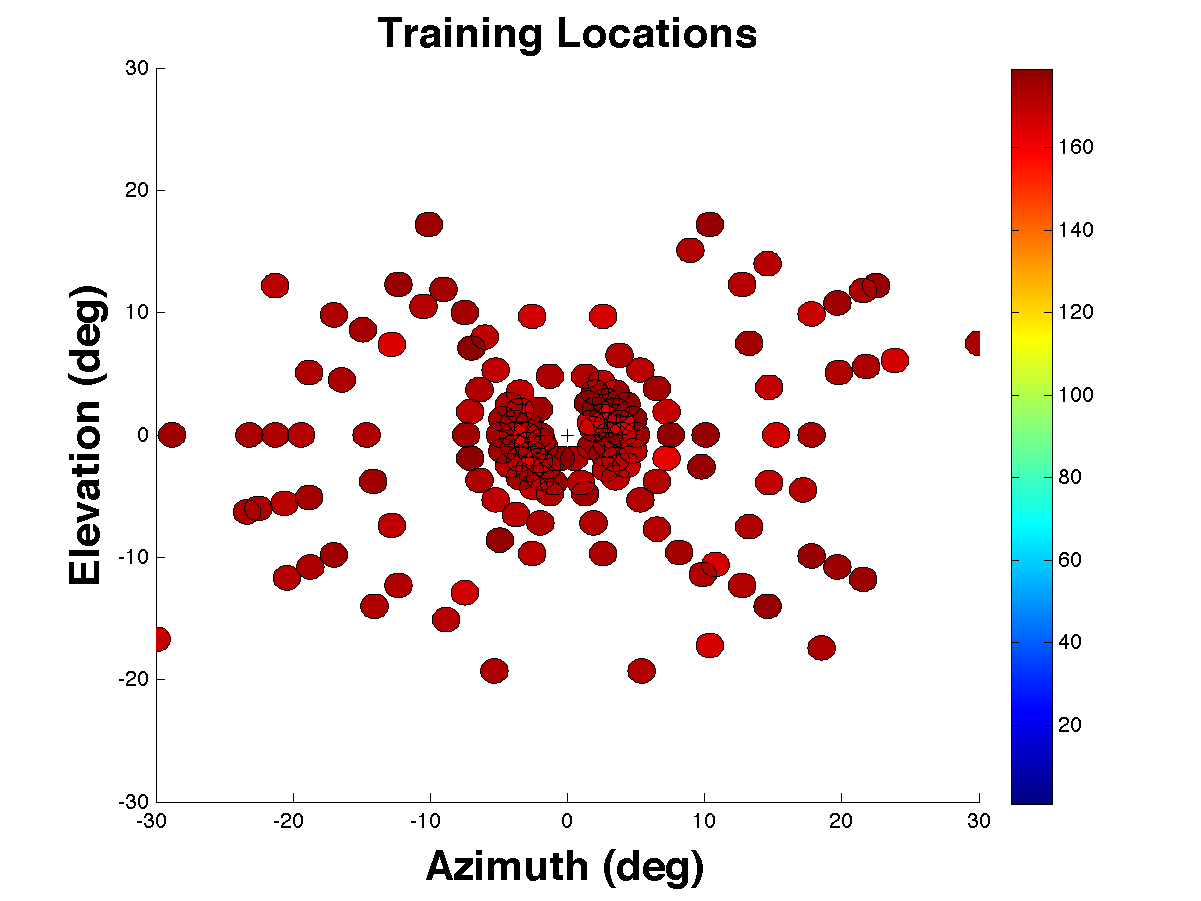 |
| J03* | M | 61 | 34 | Hemi-R | left occipital cortex | Ischemic stroke | FID | 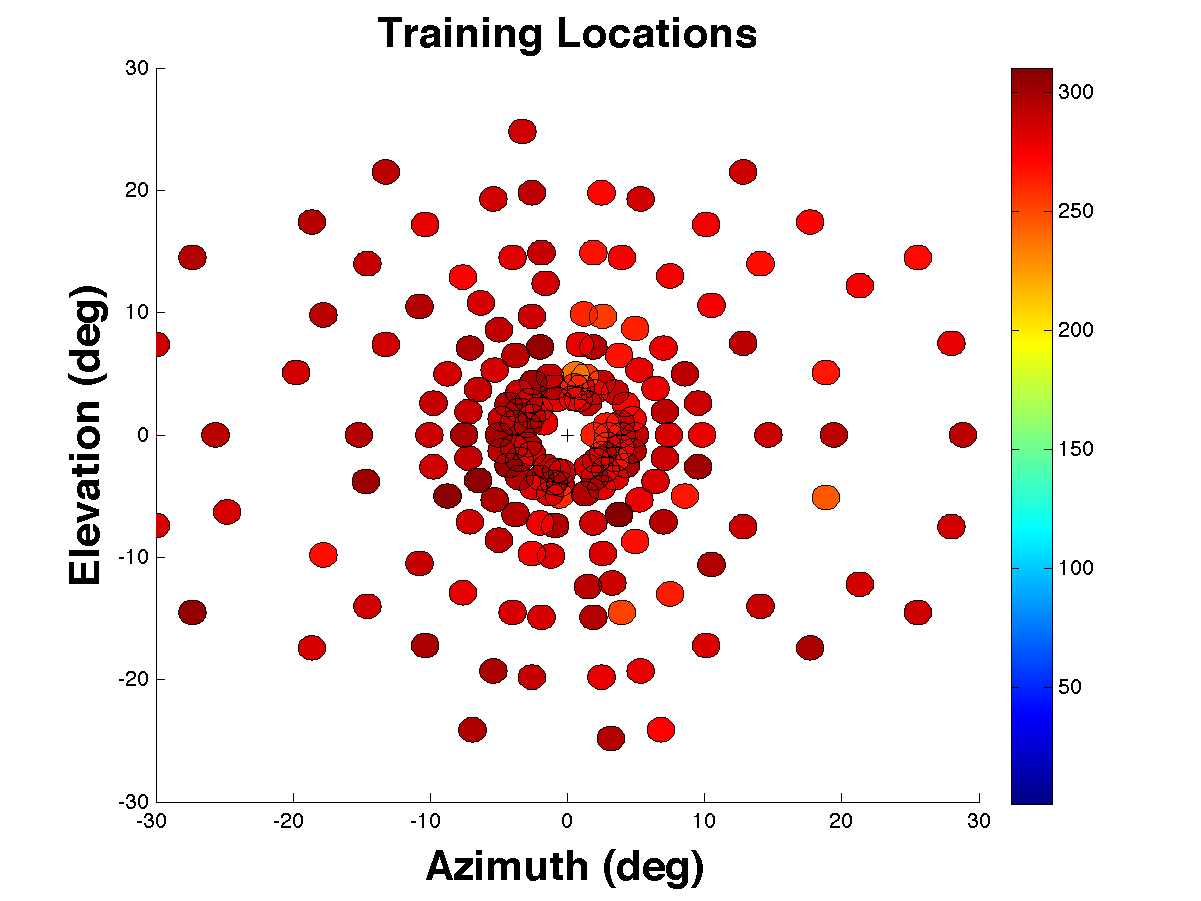 |
| J04* | M | 31 | 26 | Hemi-L  (incomplete) | right occipital cortex | Ischemic stroke | FID | 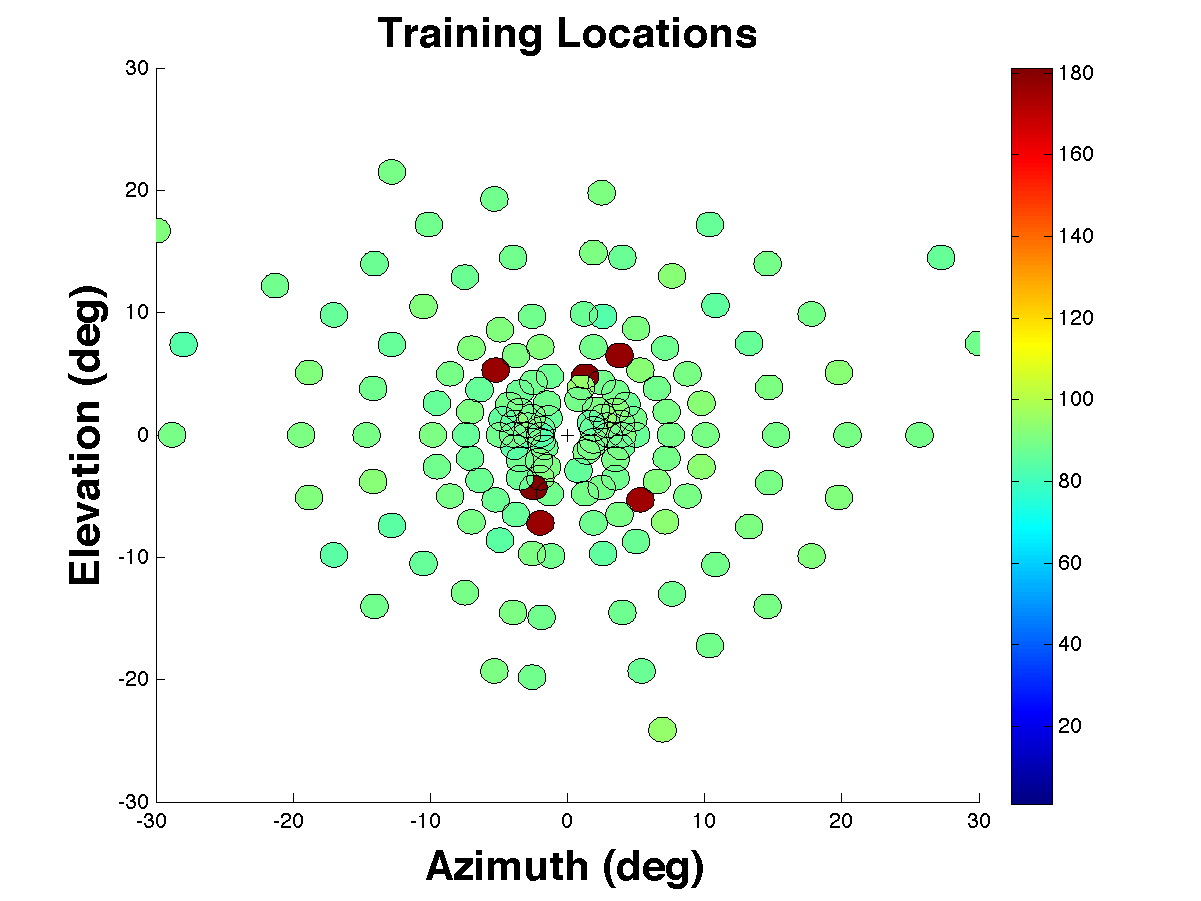 |
| J05 | M | 56 | 18 | Quadr-low-R | left occipital/parietal cortex | Ischemic stroke | FDI | 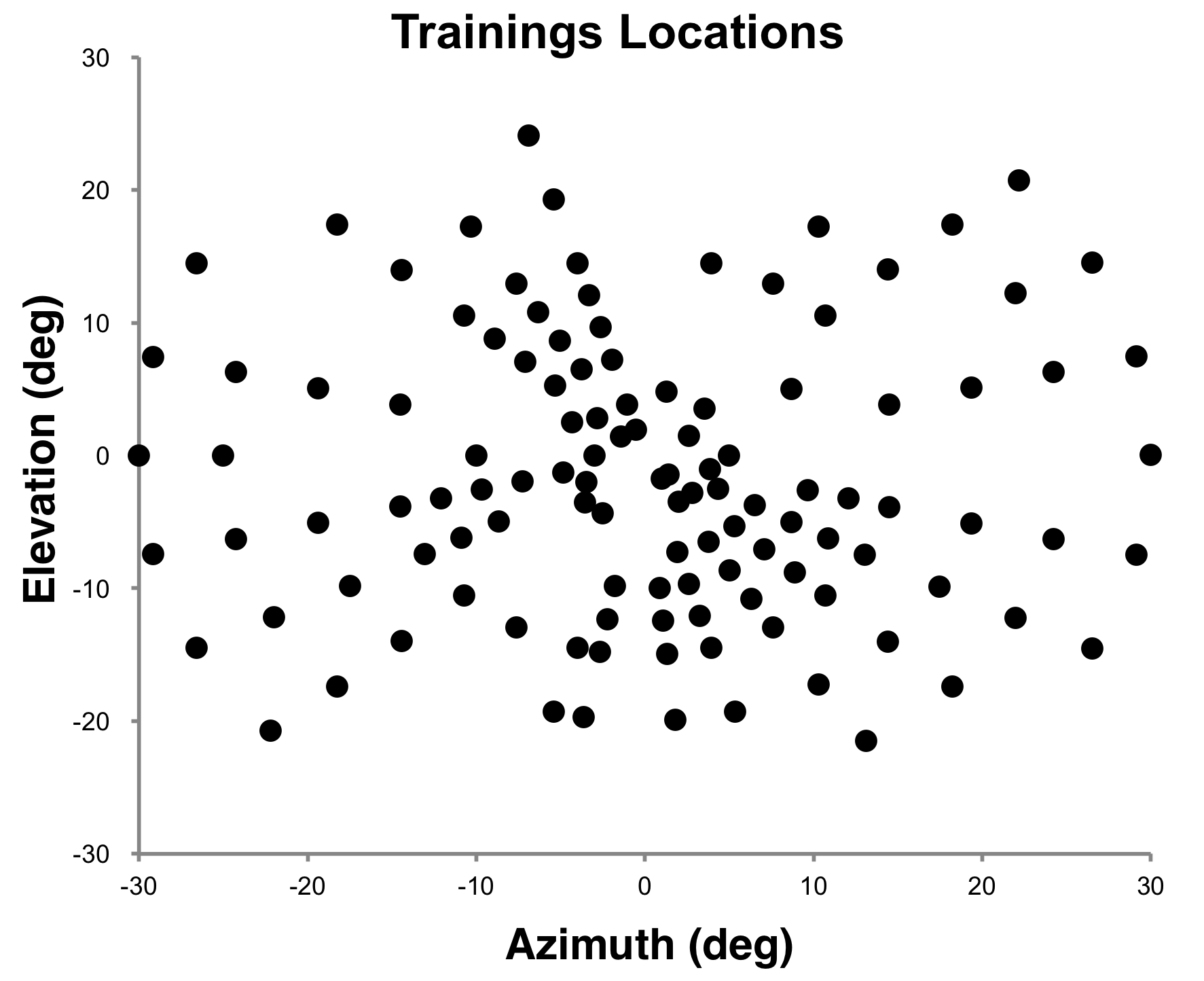 |
| J06 | M | 69 | 17 | Hemi-R  (incomplete) | left occipital cortex | Ischemic stroke | PDI | 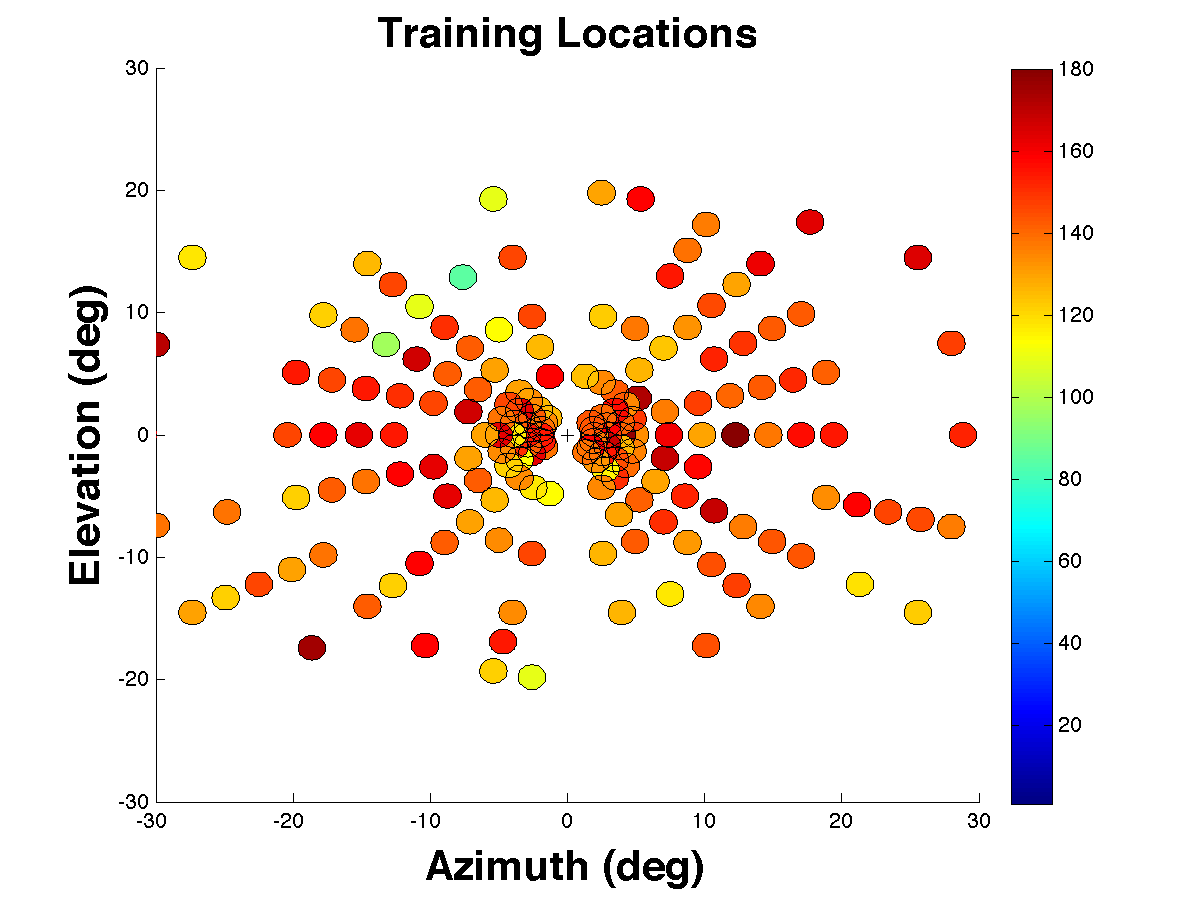 |
| J07* | F | 43 | 20 | Hemi-R  (incomplete) | left occipital cortex | Ischemic stroke | PID | 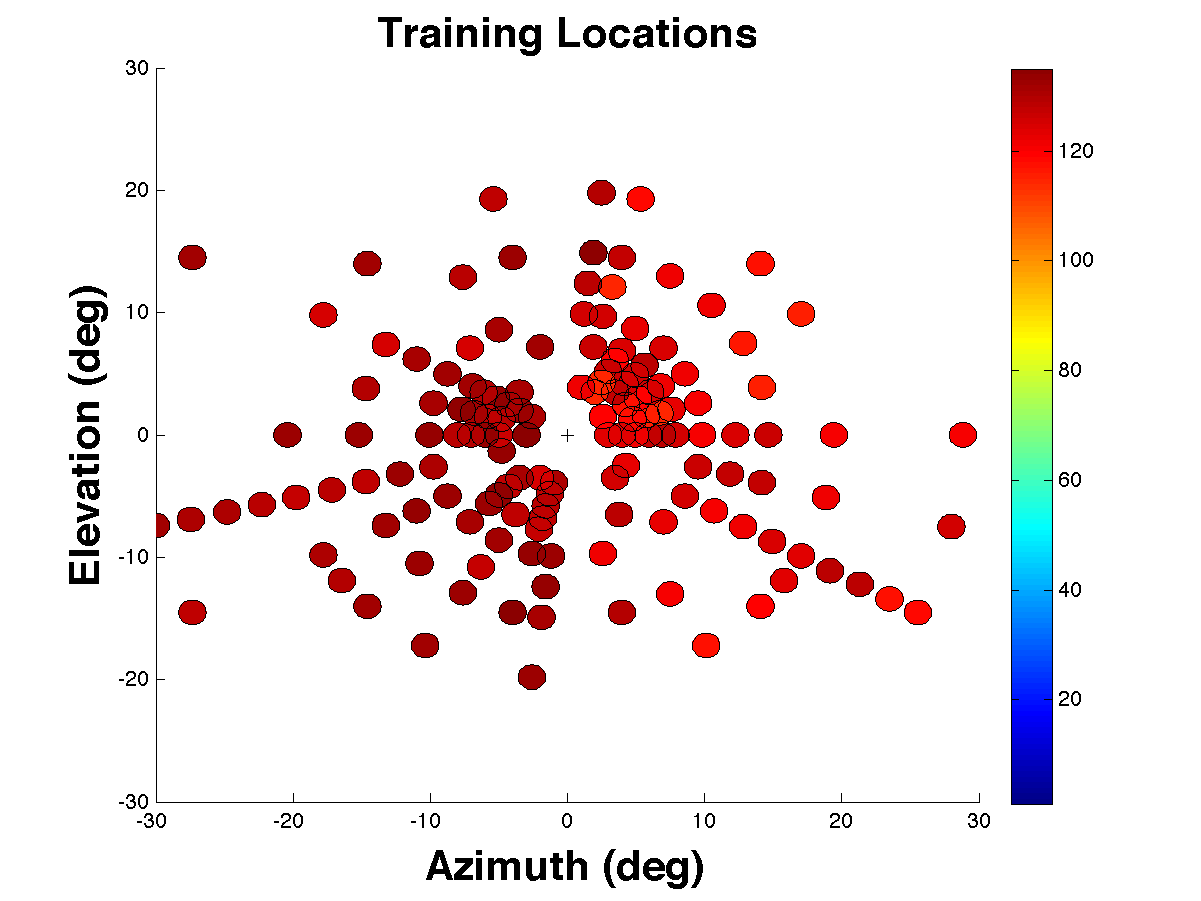 |
| J08* | F | 57 | 17 | Hemi-R | left occipital cortex | Ischemic stroke | FDI | 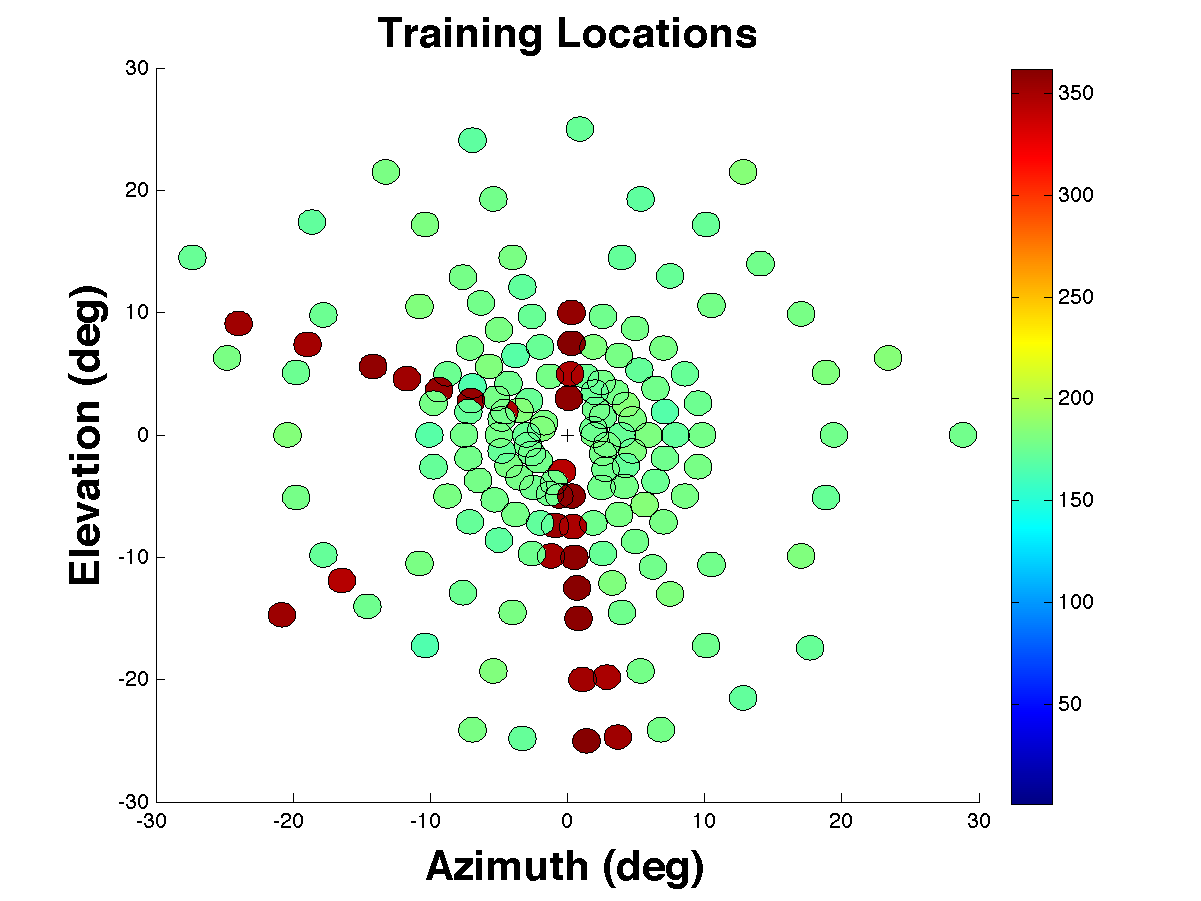 |
| J09* | M | 62 | 21 | Hemi-L  (incomplete) | right occipital cortex | Ischemic stroke | PDI | 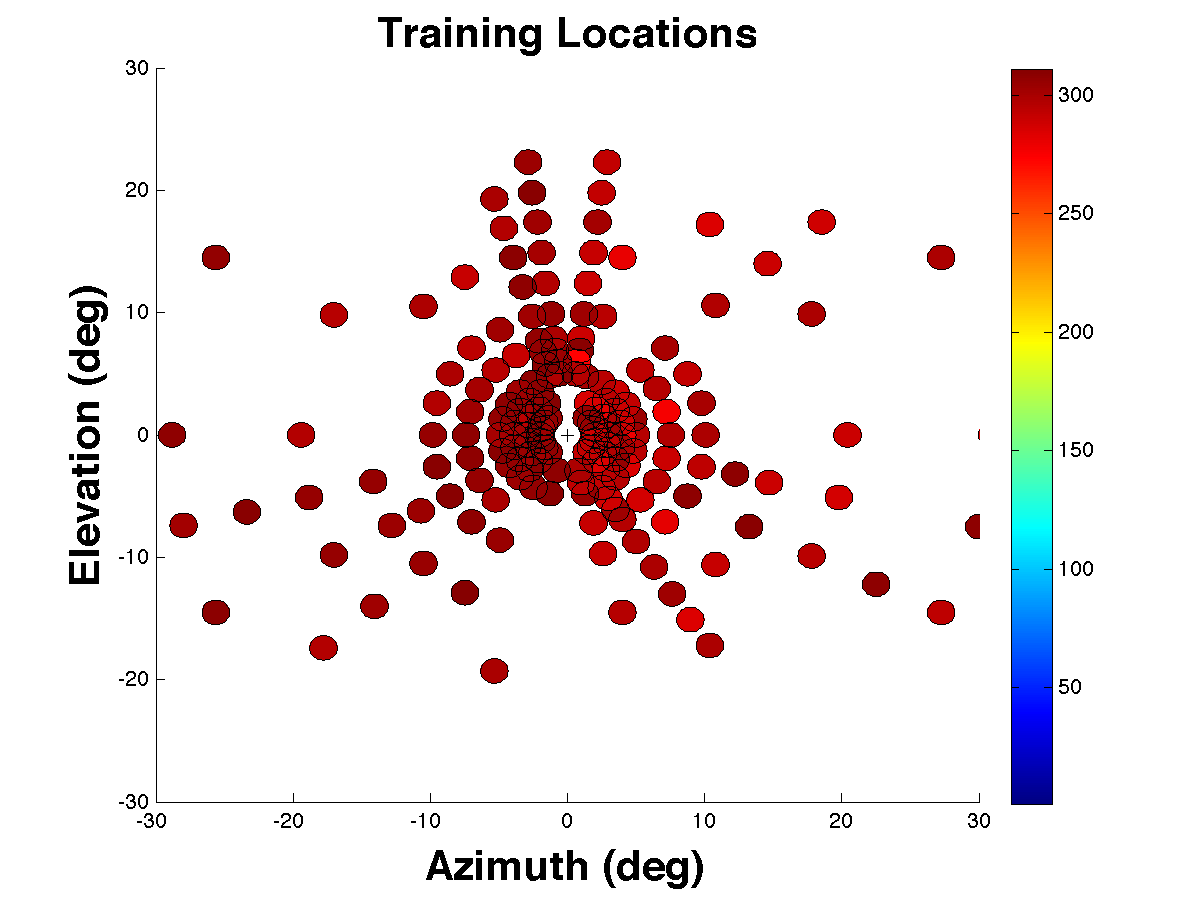 |
| J10 | M | 52 | 19 | Hemi-L  (incomplete) | right occipital/parietal cortex | Hemorrhagic stroke | PID | 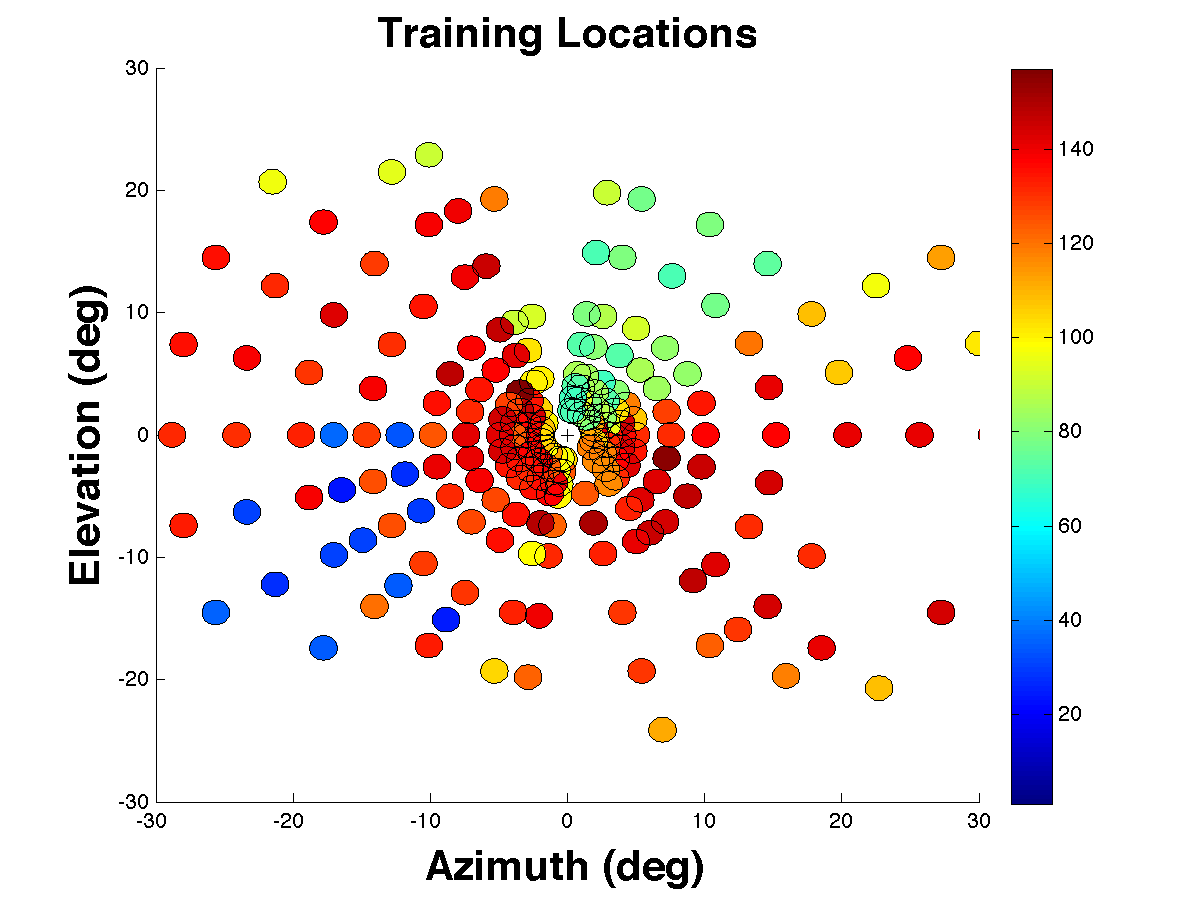 |
| J41* | F | 46 | 22 | Hemi-R  (incomplete) | left occipital cortex | Ischemic stroke | PID | 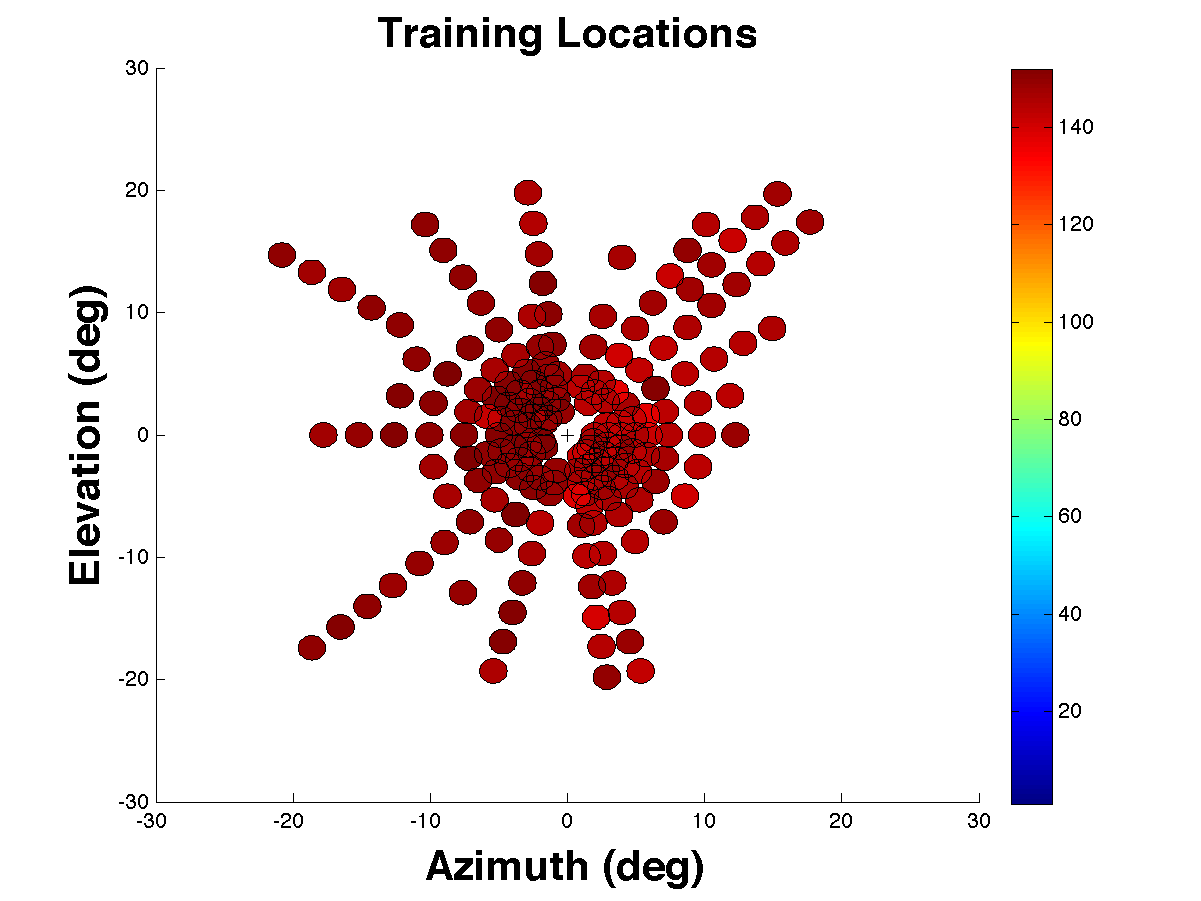 |
| J12* | M | 52 | 29 | Hemi-L  (incomplete) | right occipital cortex | Ischemic stroke | FDI | 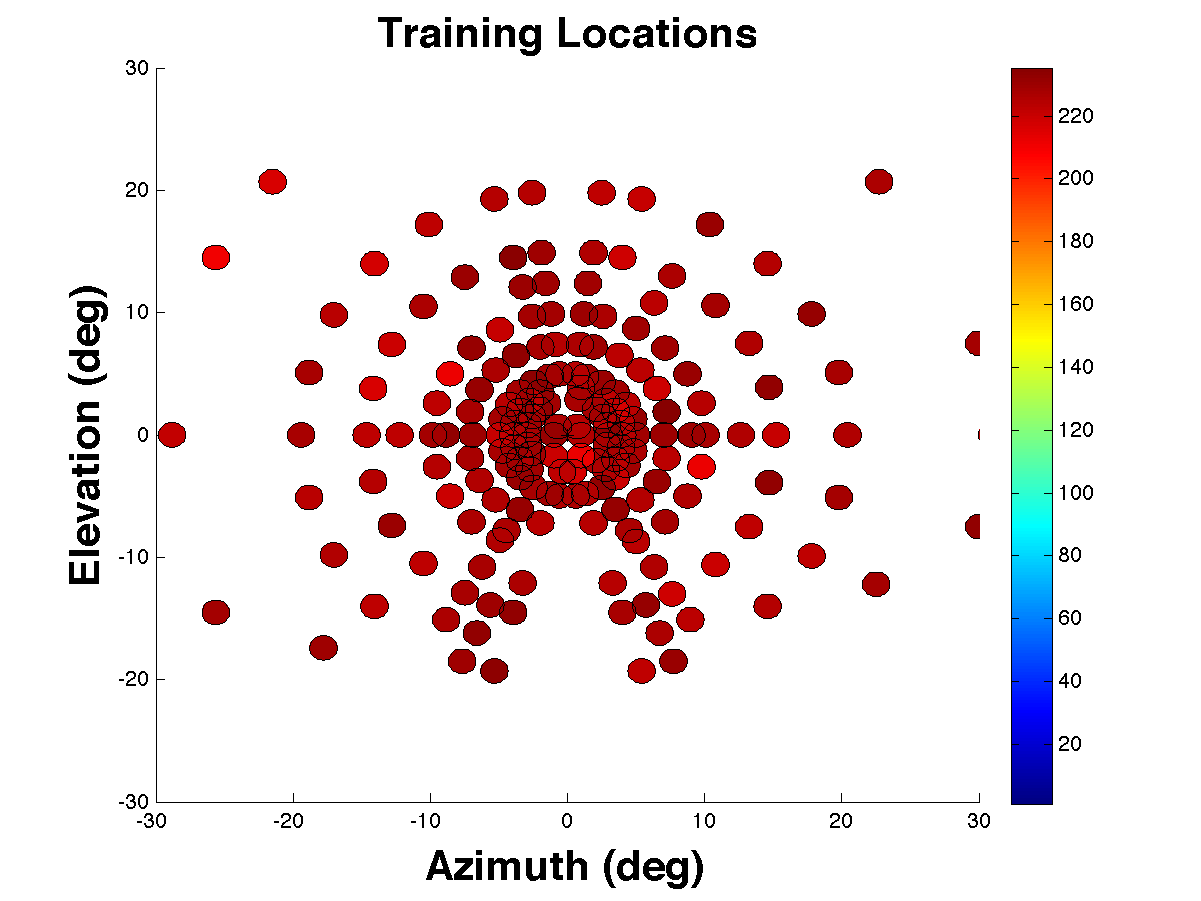 |
| J13* | M | 66 | 20 | Hemi-L | right occipital cortex | Ischemic stroke | PDI | 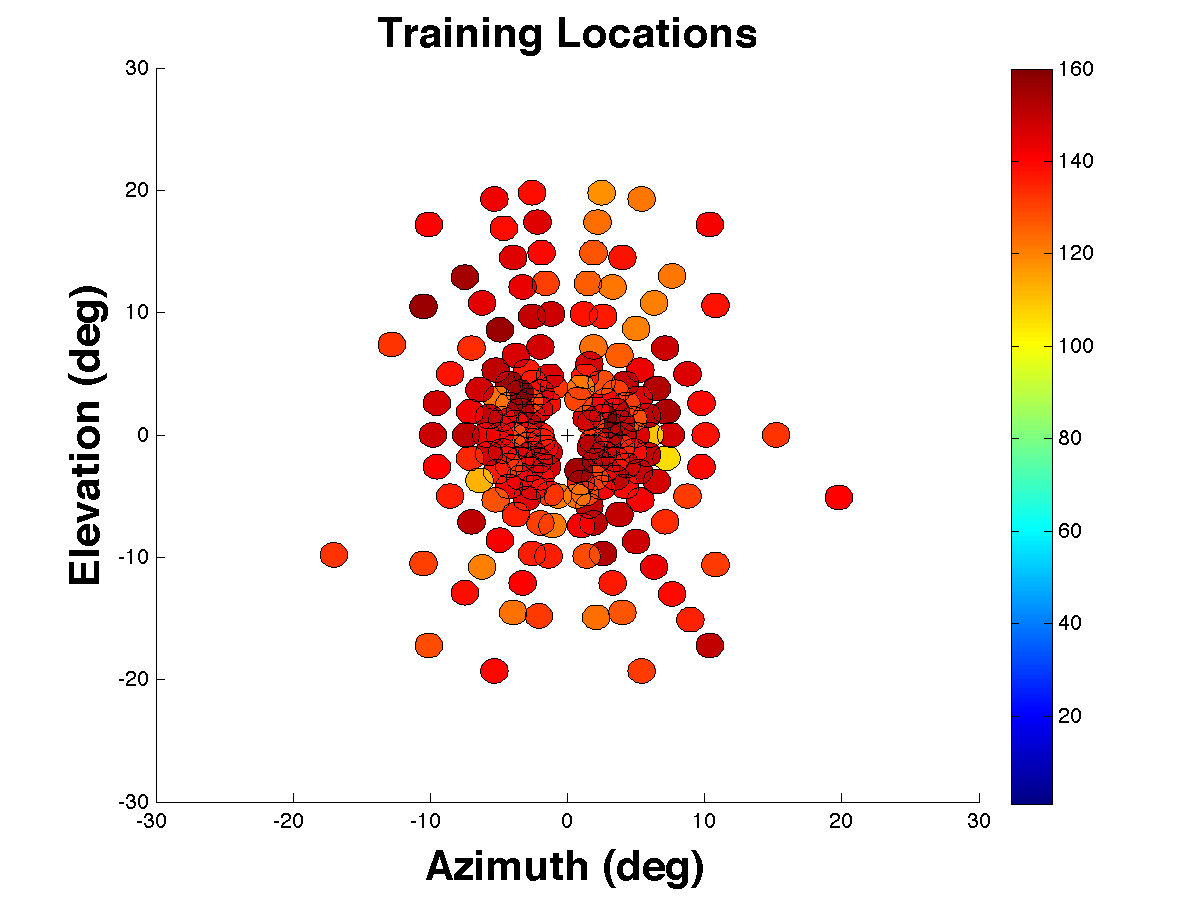 |
| J14* | M | 54 | 16 | Quadr-low-R | left occipital cortex | Ischemic stroke | FID | 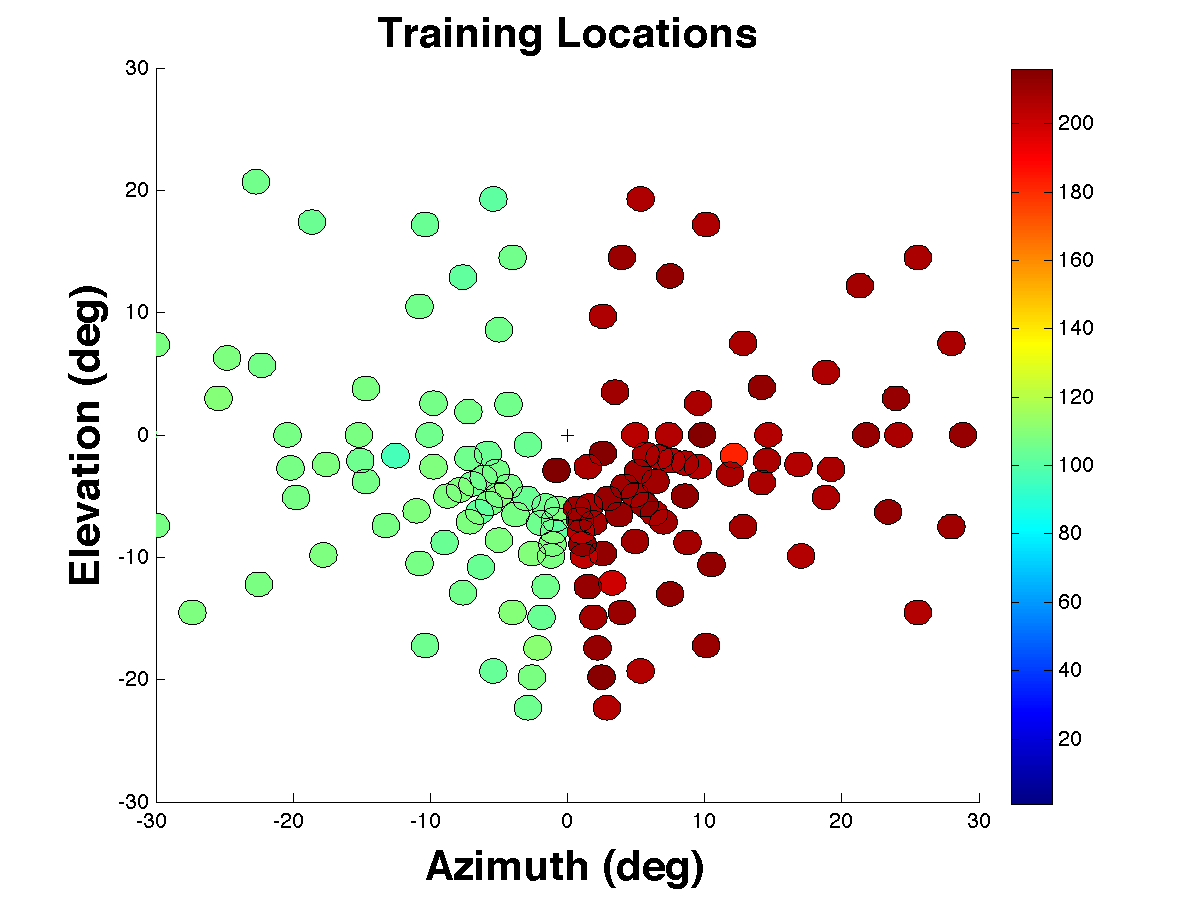 |
| J15** | M | 33 | 18 | Hemi-R | left temporal cortex/optic radiation | Hemorrhagic stroke | FDI | 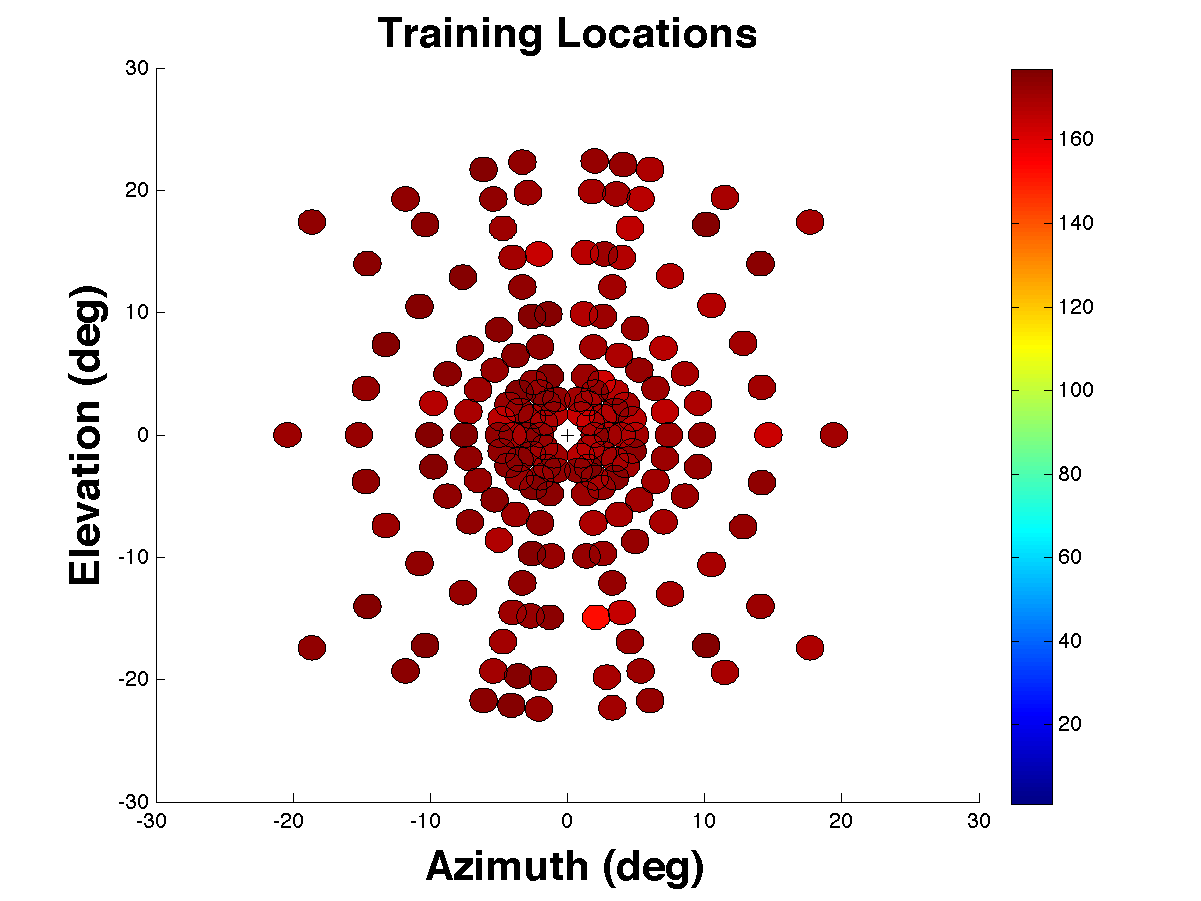 |
| J16** | F | 43 | 21 | Hemi-R  (incomplete) | left occipital cortex | Ischemic stroke | PID | 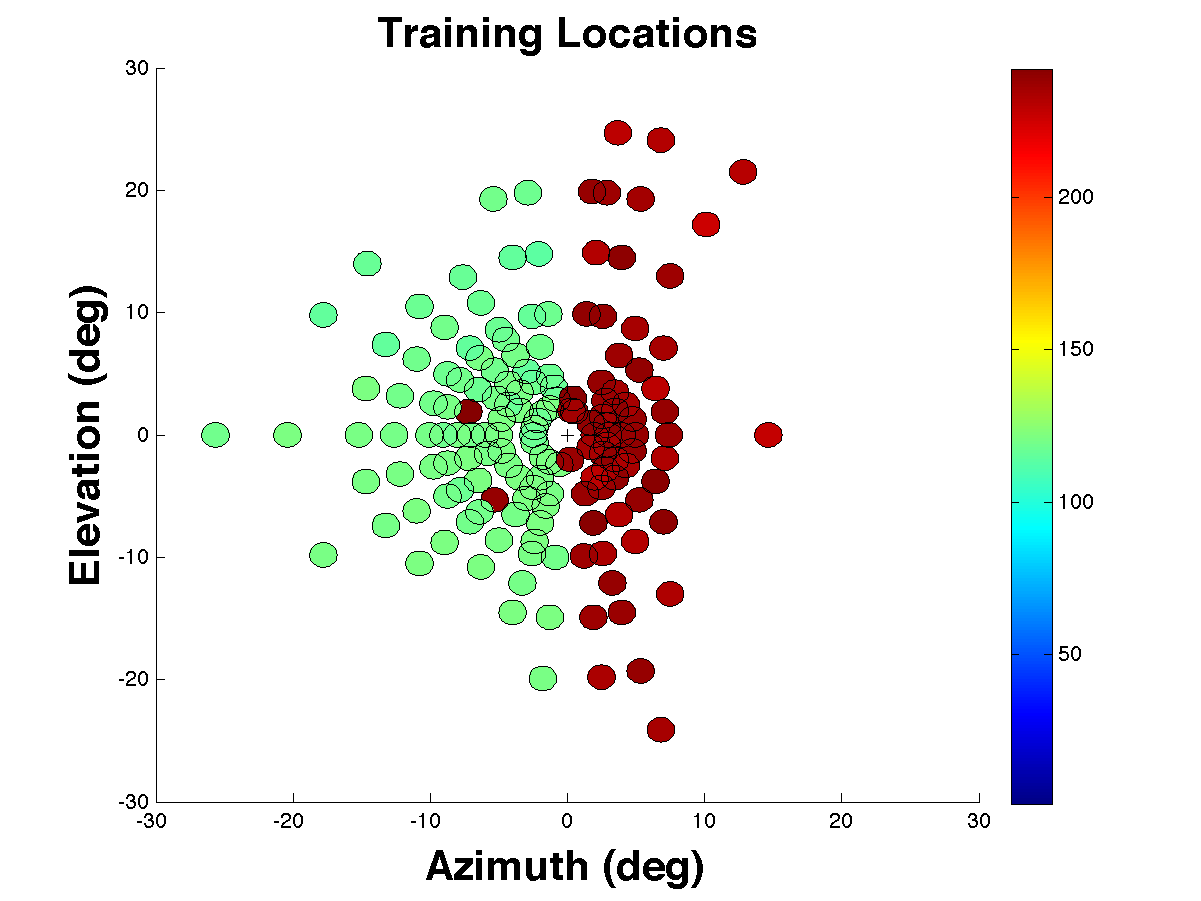 |
| J17* | M | 43 | 19 | Bilateral incomplete hemianopia | right and left occipital cortex | Ischemic stroke | PID | 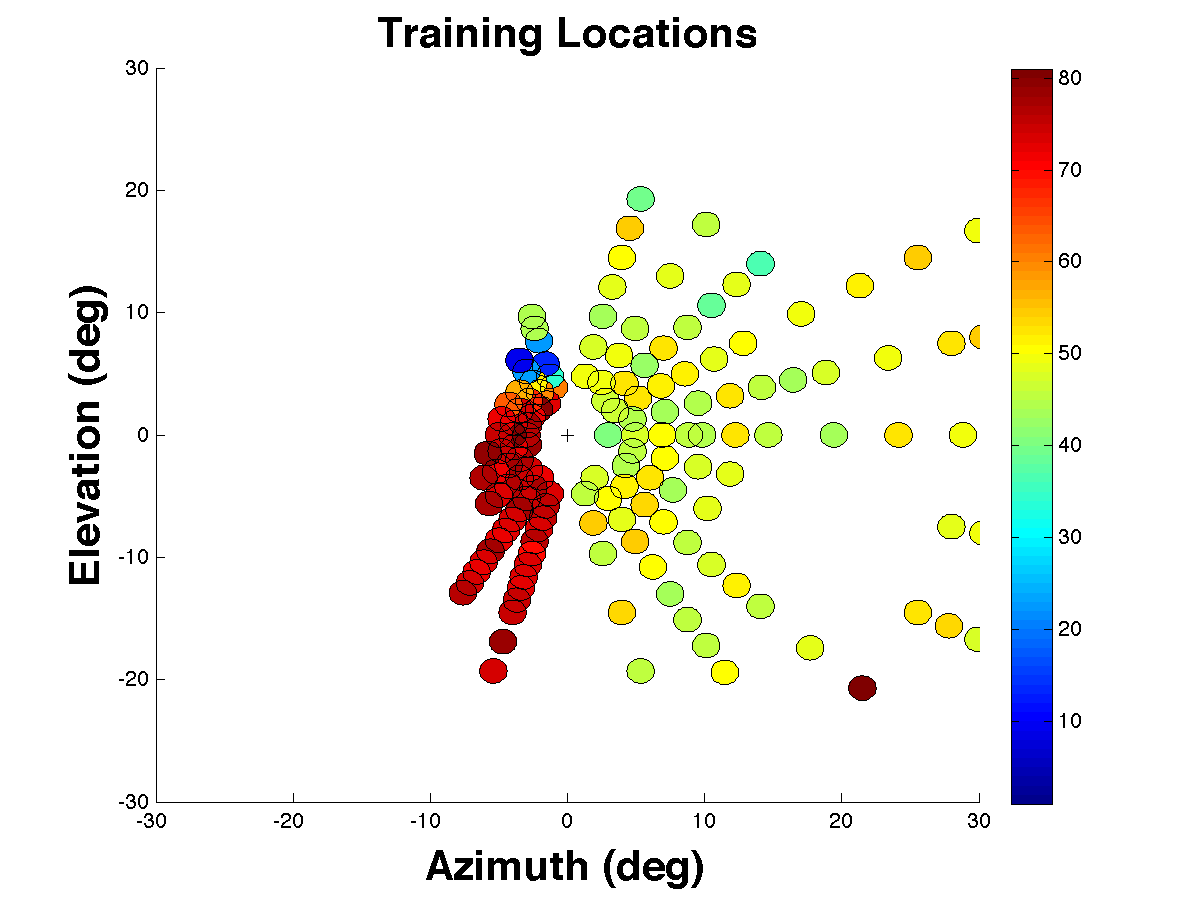 |
| J18* | M | 48 | 17 | Scot-up-L | right occipital cortex | Ischemic stroke | FDI | 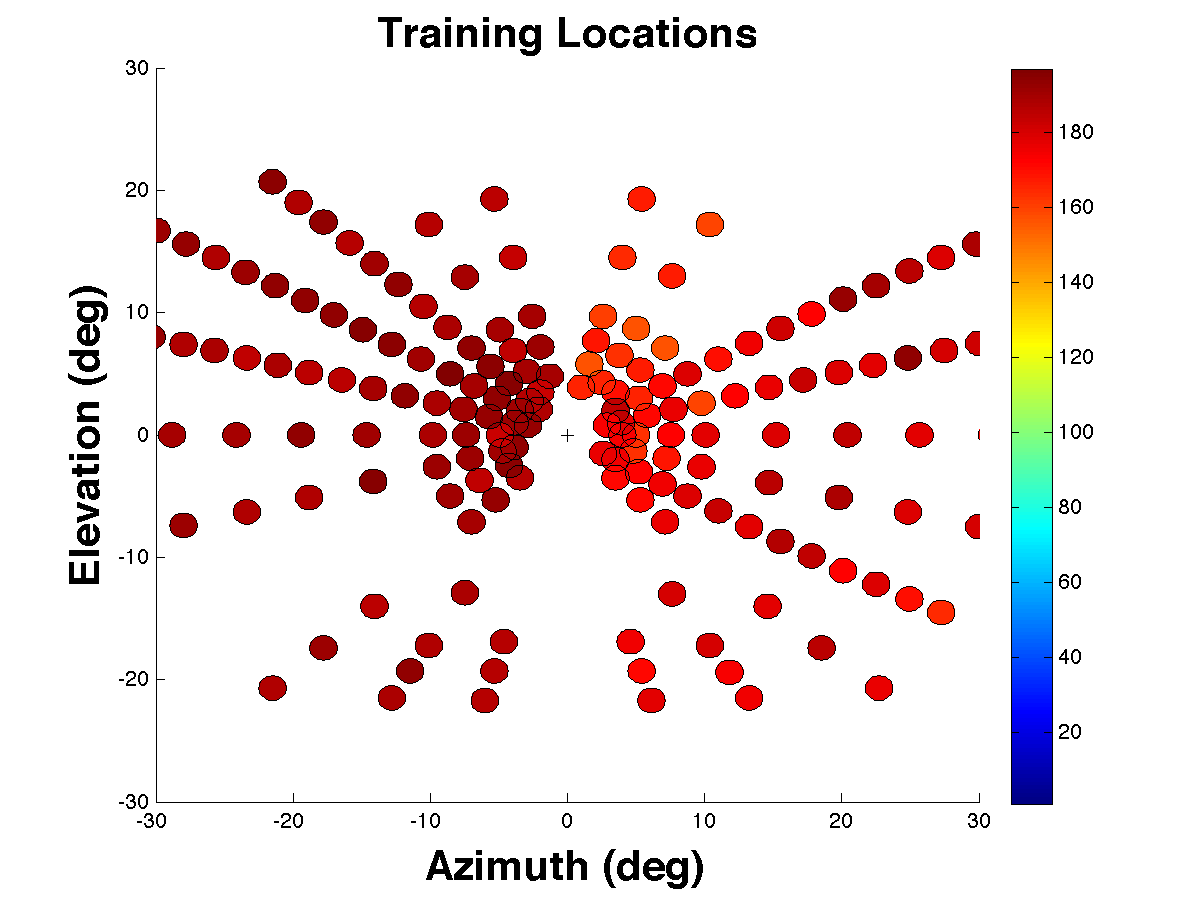 |
| J20 | M | 53 | 17 | Hemi-L  (incomplete) | right temporal/parietal cortex (optic radiation) | Ischemic stroke | PDI | 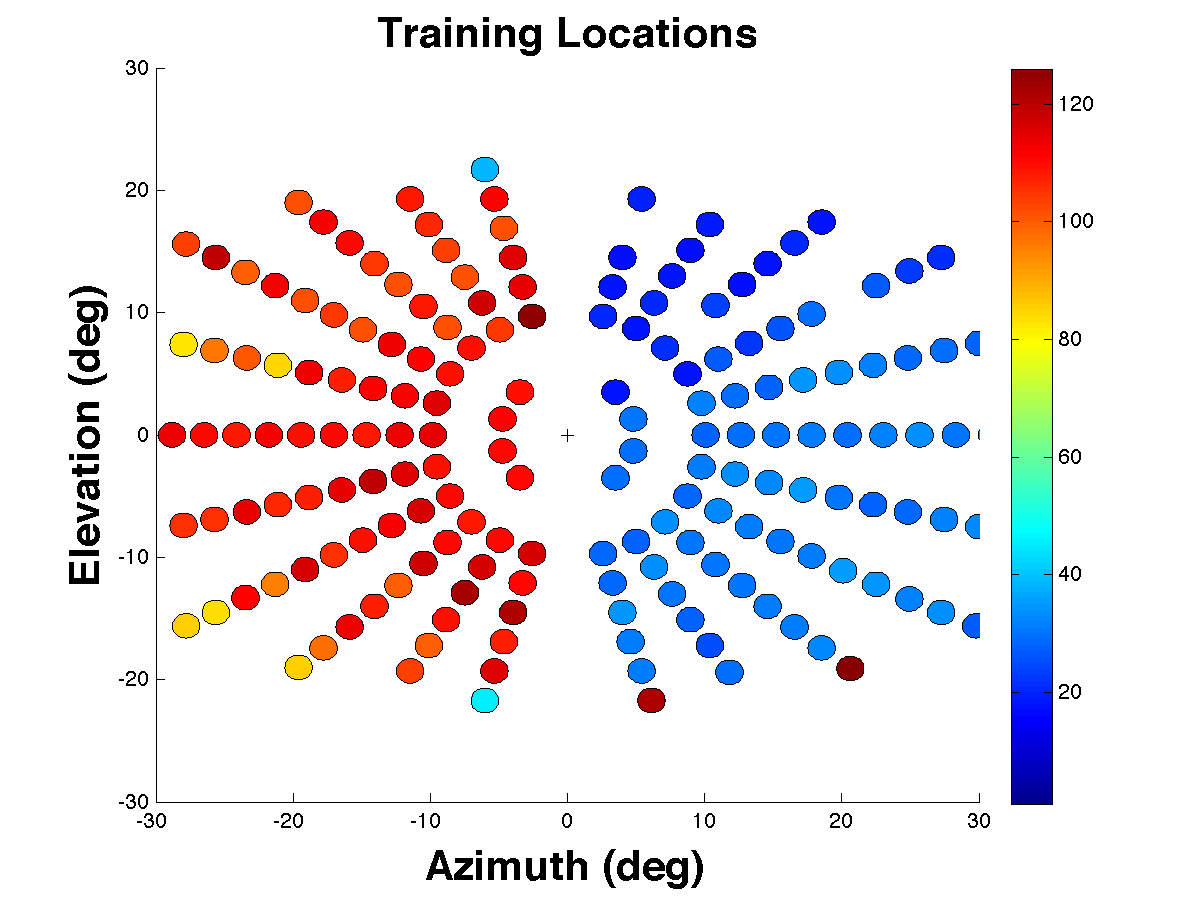 |
| J22* | M | 74 | 17 | Hemi-L  (incomplete) | right occipital cortex | Ischemic stroke | PDI | 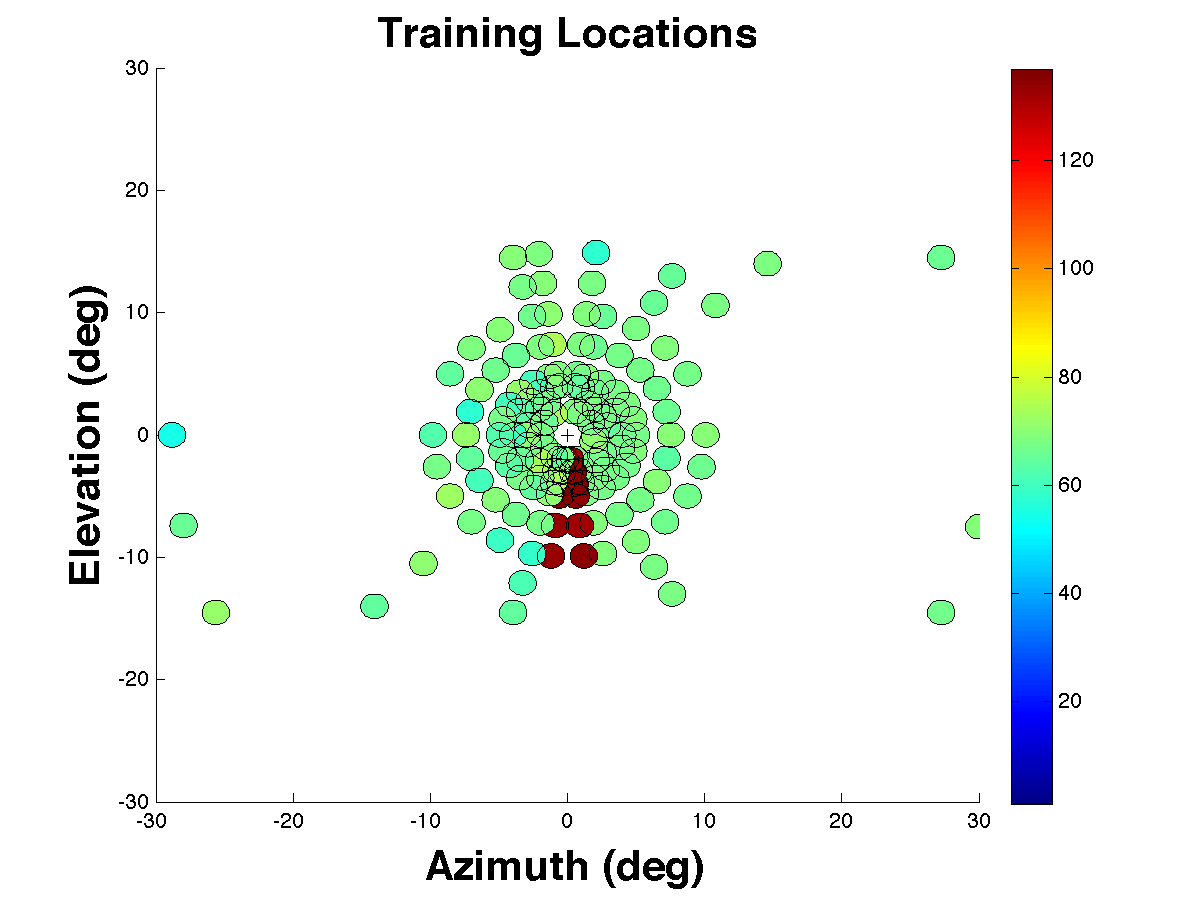 |
| J23* | M | 39 | 85 | Scot-R | left occipital cortex | Hemorrhagic stroke | FDI | 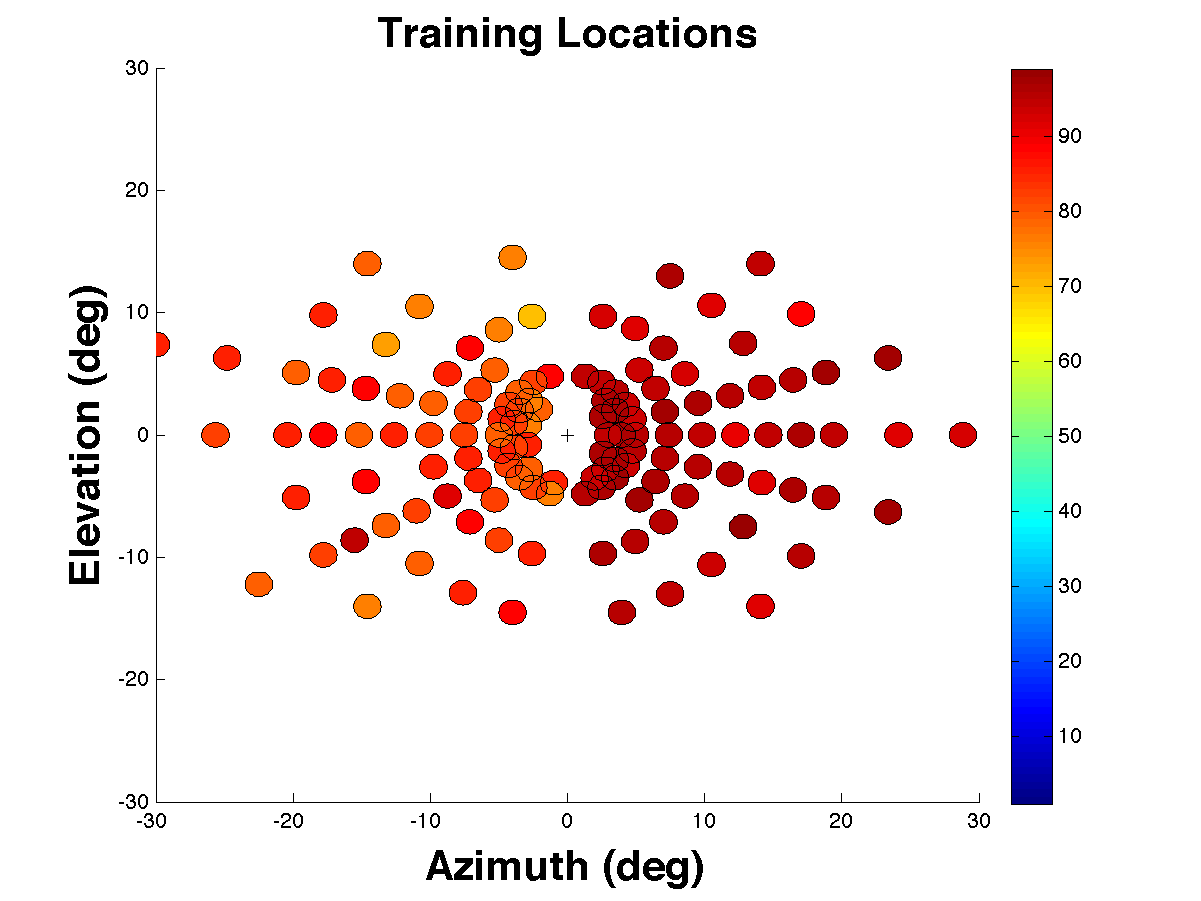 |
| J24 | F | 46 | 111 | Hemi-L  (incomplete) | right occipital cortex | Ischemic stroke | FID | 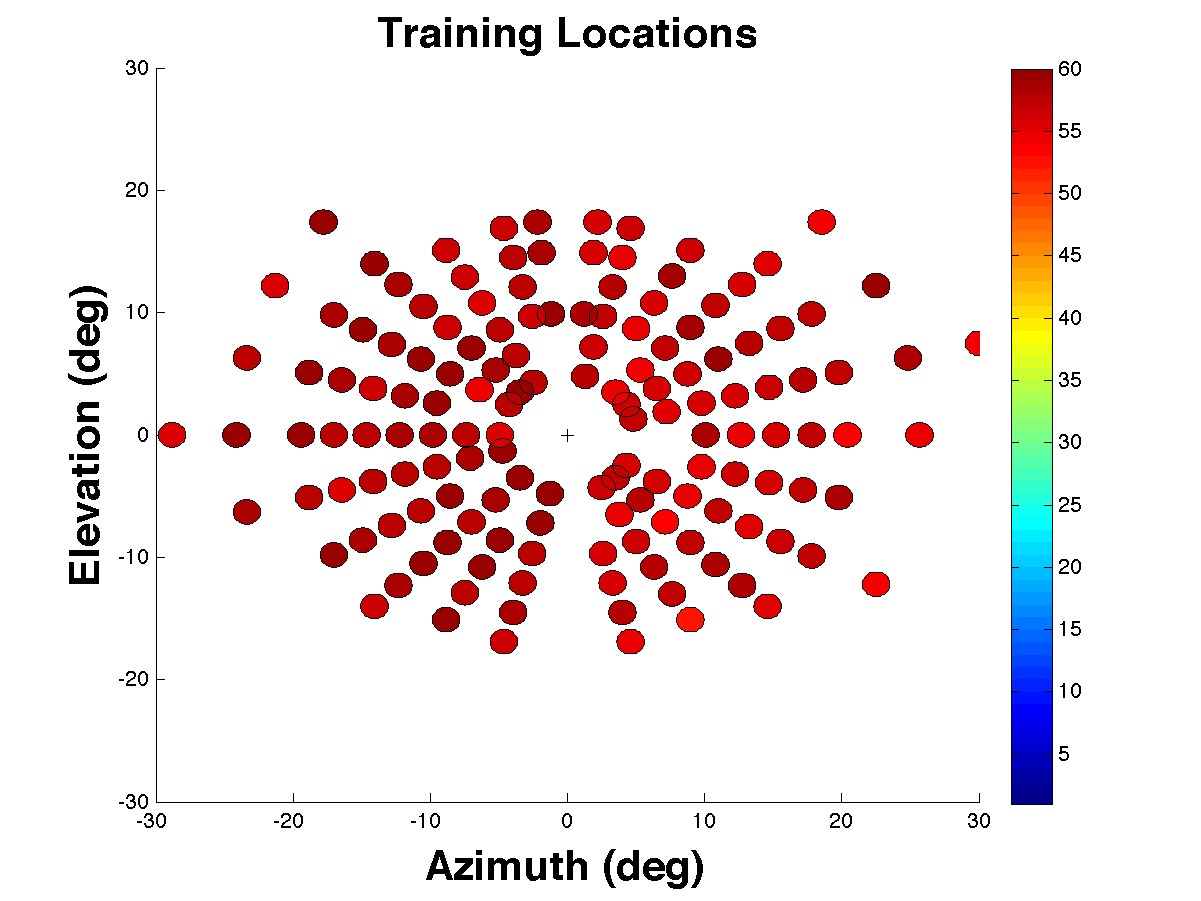 |
| J25 | M | 48 | 19 | Quadr-up-L | right occipital cortex | Ischemic stroke | FID | 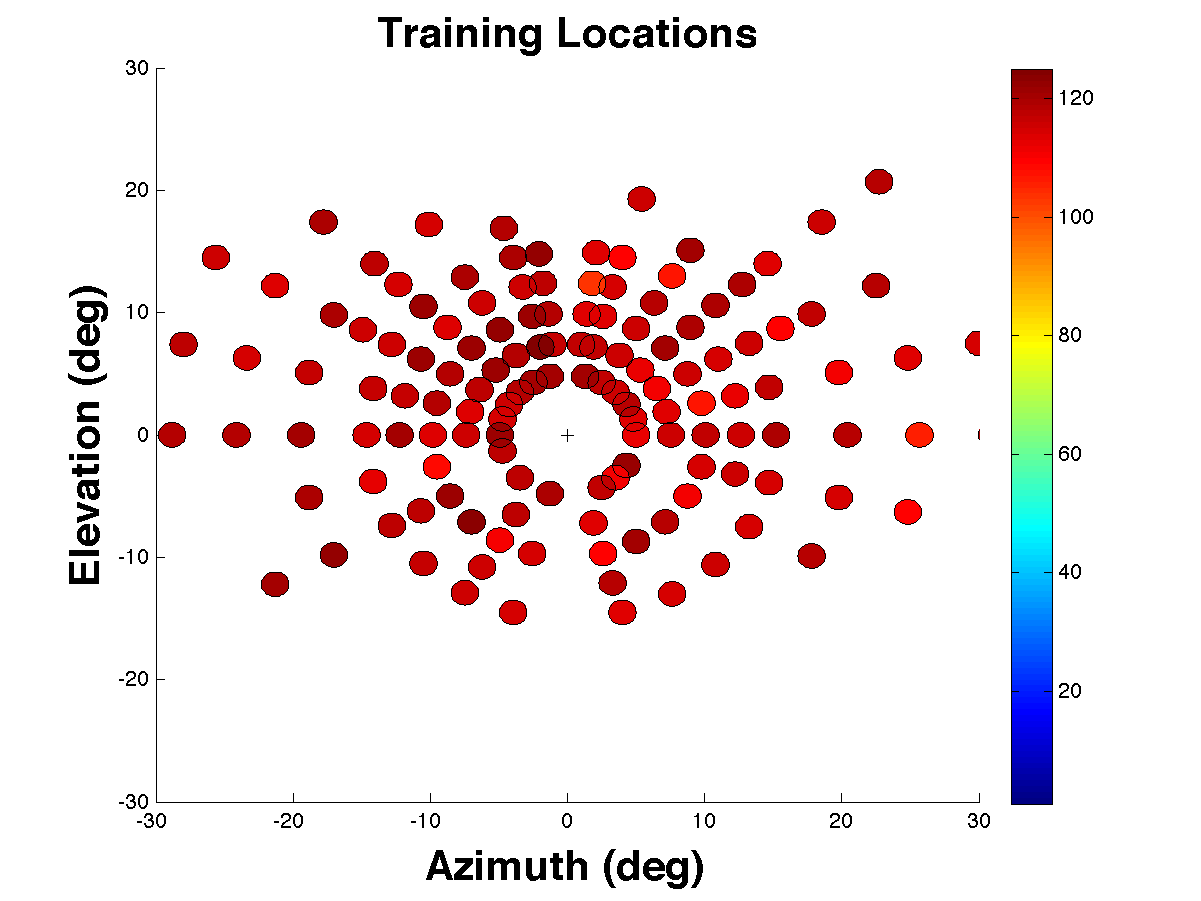 |
| J26* | M | 29 | 21 | Hemi-R  (incomplete) | left occipital cortex | Ischemic stroke | PDI | 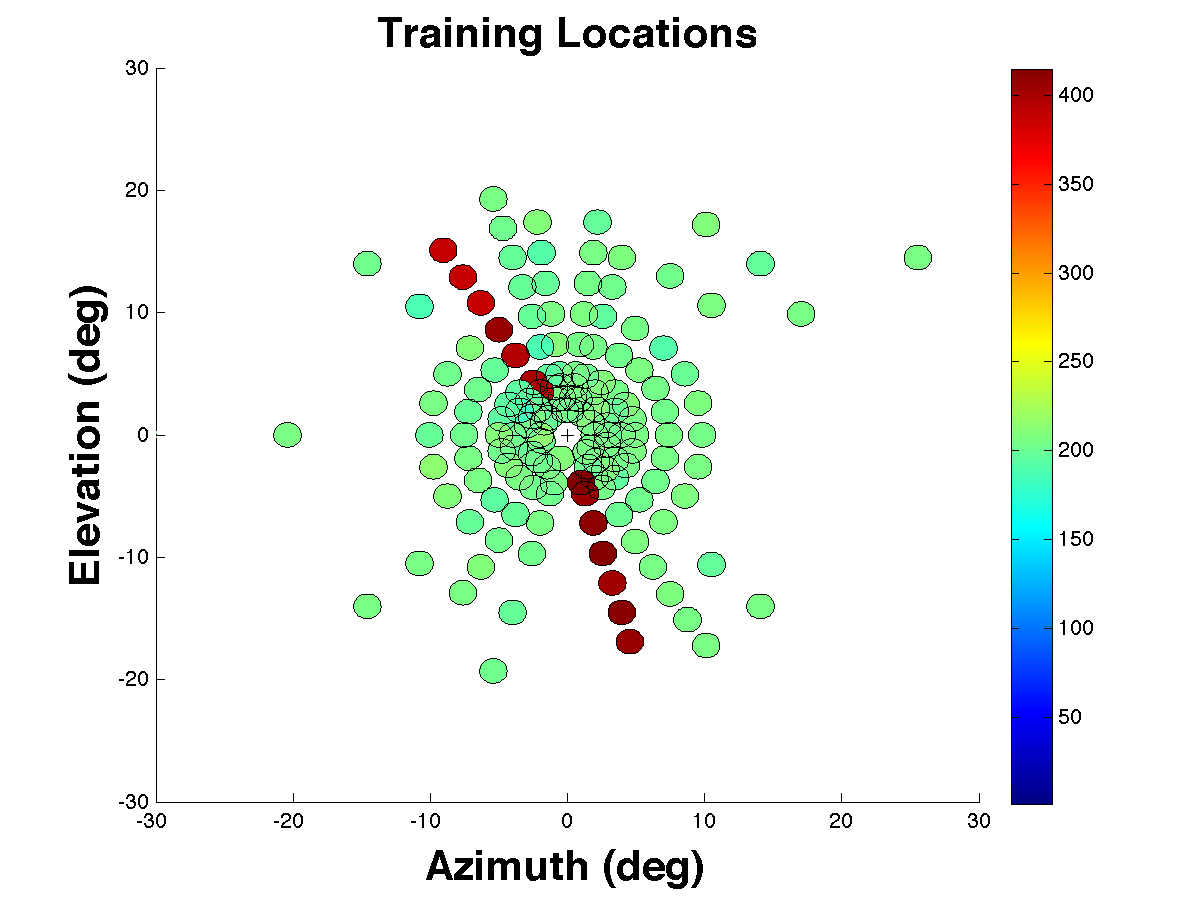 |
| J27 | M | 49 | 38 | Hemi-R | Left occipital/temporal cortex | Hemorrhagic stroke | FDI | 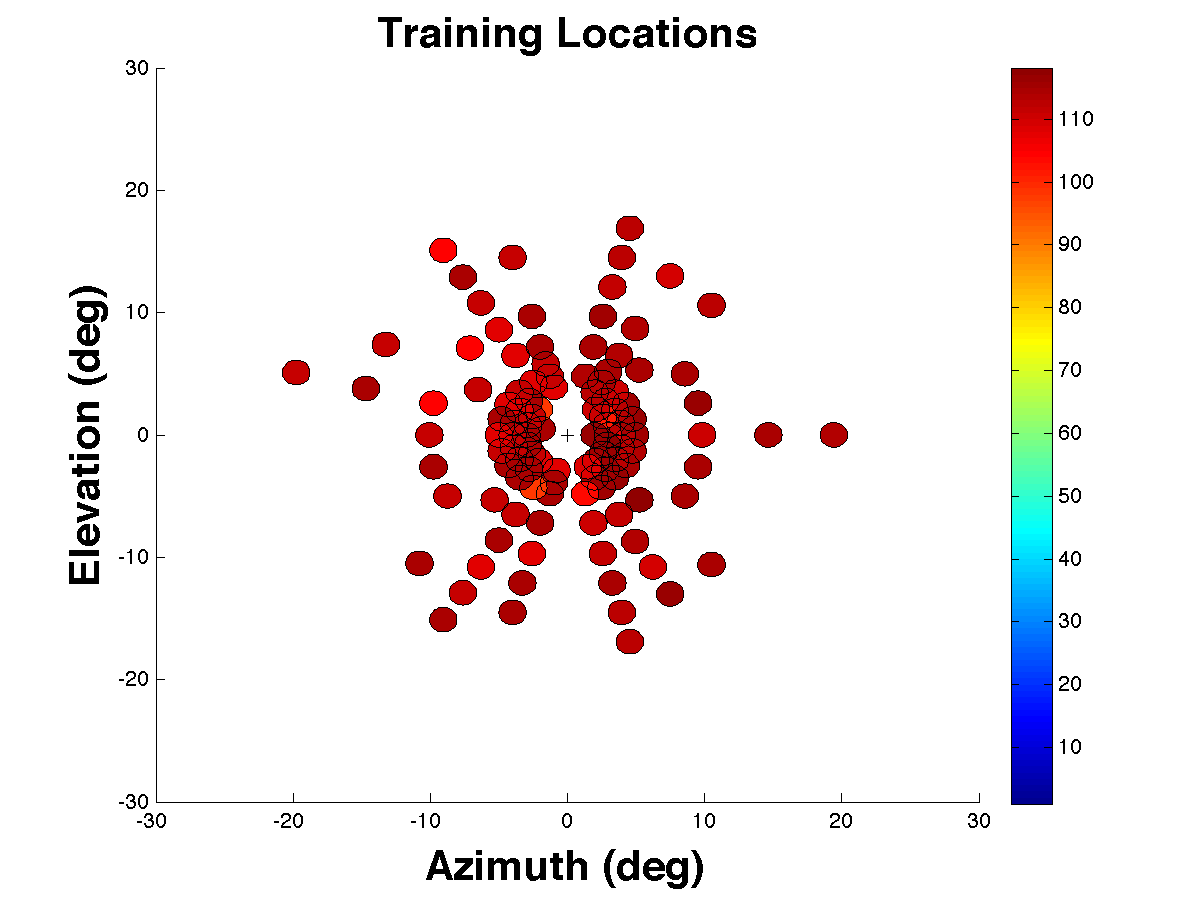 |
| J28 | M | 68 | 19 | Hemi-L  (incomplete) | right occipital cortex | Ischemic stroke | FID | 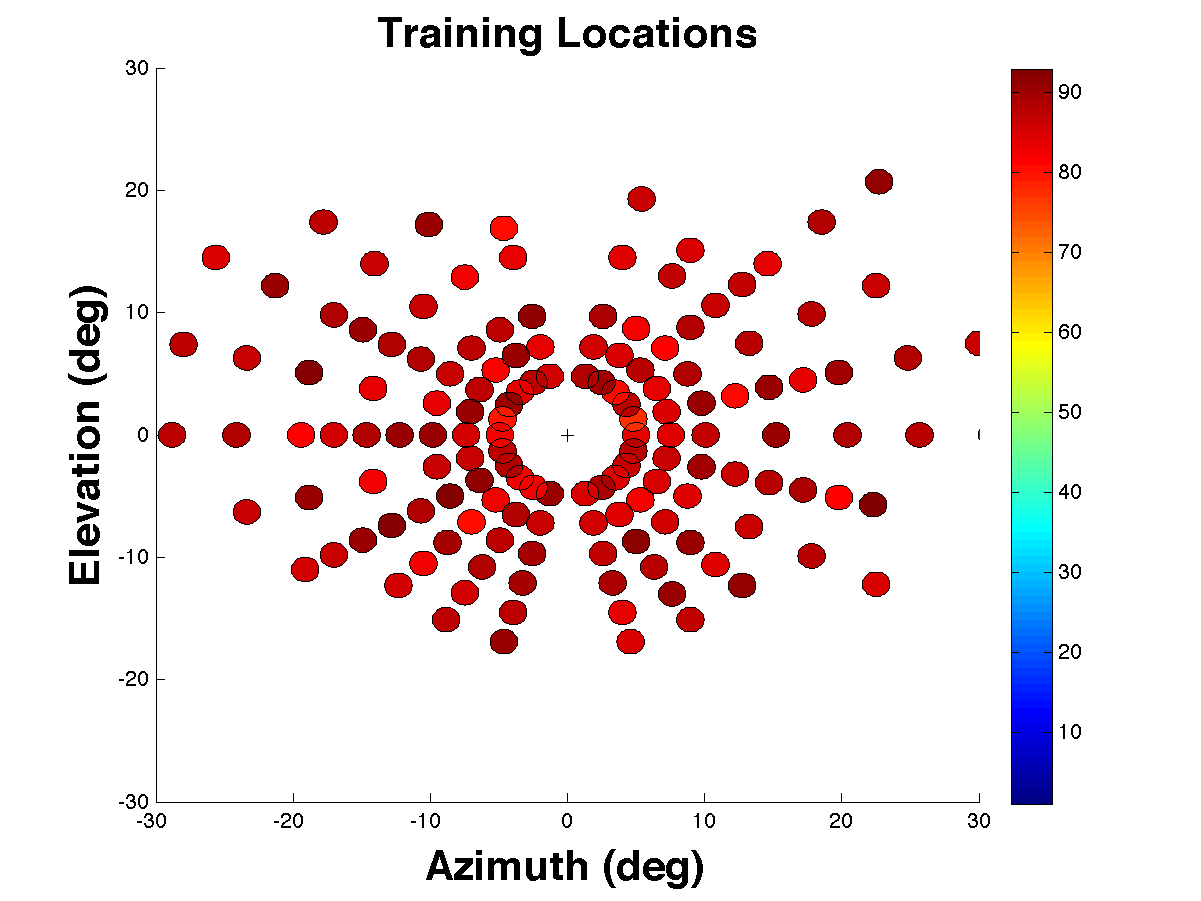 |
| J30** | M | 34 | 11 | Hemi-L  (incomplete) | right occipital/parietal cortex | Hemorrhagic stroke | PID | 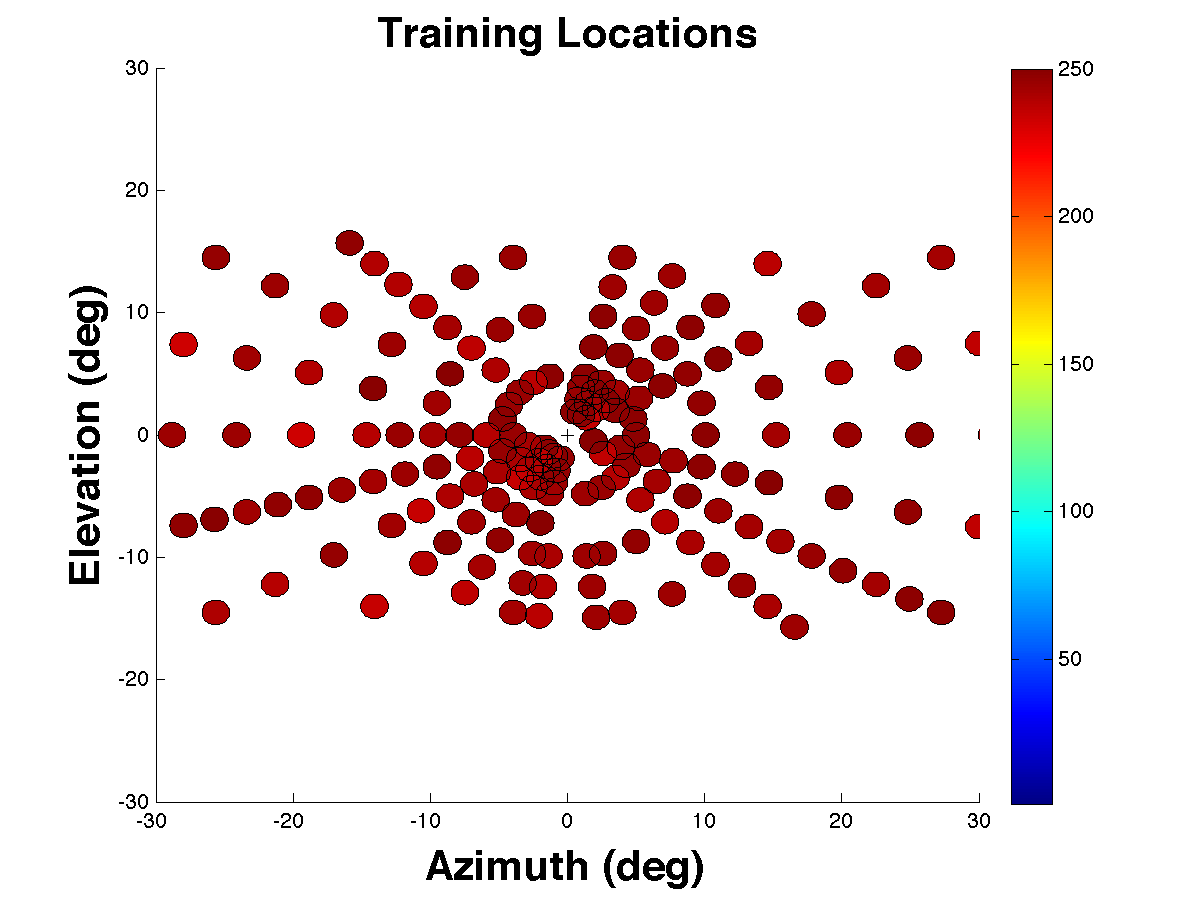 |

* >50% of home training data analyzed; ** >90% of home training data analyzed

^a^ FID = flow stimulus, intact training first; FDI = flow stimulus, defect training first; PID = point stimulus, intact training first; PDI = point stimulus, defect training first
